# Metabolic reprogramming in placenta and umbilical cord serum of polycystic ovary syndrome pregnancies: testosterone-associated alterations and interaction with obesity

**DOI:** 10.1093/hropen/hoag036

**Published:** 2026-04-21

**Authors:** Huisheng Ge, Xiafei Wu, Dongni Huang, Li He, Dandan Liu, Lulu Wang, Liling Xiong, Dan Luo, Qiannan Hou, Hong Liu, Lunbo Tan, Yonghong Lin, Chang Chen, Xixi Wu

**Affiliations:** Department of Gynecology and Obstetrics, Chengdu Women’s and Children’s Central Hospital, School of Medicine, University of Electronic Science and Technology of China, Chengdu, China; Shanghai Key Laboratory of Maternal-Fetal Medicine, Department of Biobank, Clinical and Translational Research Center, Shanghai First Maternity and Infant Hospital, School of Medicine, Tongji University, Shanghai, China; Department of Obstetrics and Gynecology, Women and Children’s Hospital of Chongqing Medical University (Chongqing Health Center for Women and Children), Chongqing, China; Chongqing Key Laboratory of Maternal and Fetal Medicine, Chongqing Medical University, Chongqing, China; Department of Gynecology and Obstetrics, Chengdu Women’s and Children’s Central Hospital, School of Medicine, University of Electronic Science and Technology of China, Chengdu, China; Department of Gynecology and Obstetrics, Chengdu Women’s and Children’s Central Hospital, School of Medicine, University of Electronic Science and Technology of China, Chengdu, China; Department of Gynecology and Obstetrics, Chengdu Women’s and Children’s Central Hospital, School of Medicine, University of Electronic Science and Technology of China, Chengdu, China; Department of Gynecology and Obstetrics, Chengdu Women’s and Children’s Central Hospital, School of Medicine, University of Electronic Science and Technology of China, Chengdu, China; Department of Gynecology and Obstetrics, Chengdu Women’s and Children’s Central Hospital, School of Medicine, University of Electronic Science and Technology of China, Chengdu, China; Department of Gynecology and Obstetrics, Chengdu Women’s and Children’s Central Hospital, School of Medicine, University of Electronic Science and Technology of China, Chengdu, China; Department of Gynecology and Obstetrics, Chengdu Women’s and Children’s Central Hospital, School of Medicine, University of Electronic Science and Technology of China, Chengdu, China; Department of Obstetrics and Gynecology, Women and Children’s Hospital of Chongqing Medical University (Chongqing Health Center for Women and Children), Chongqing, China; Chongqing Key Laboratory of Maternal and Fetal Medicine, Chongqing Medical University, Chongqing, China; Department of Gynecology and Obstetrics, Chengdu Women’s and Children’s Central Hospital, School of Medicine, University of Electronic Science and Technology of China, Chengdu, China; College of Pharmacy, Chongqing Medical University, Chongqing, China; Department of Gynecology and Obstetrics, Chengdu Women’s and Children’s Central Hospital, School of Medicine, University of Electronic Science and Technology of China, Chengdu, China

**Keywords:** polycystic ovary syndrome, metabolomics, lipidomics, placenta, umbilical cord serum, obesity

## Abstract

**STUDY QUESTION:**

What are the specific metabolic and lipidomic alterations in placental tissue and umbilical cord serum of pregnancies complicated by PCOS, and how do maternal testosterone levels and obesity status interplay with these changes at the maternal–fetal interface?

**SUMMARY ANSWER:**

Pregnancies affected by PCOS exhibit distinct metabolomic and lipidomic reprogramming in both placental tissue and umbilical cord serum, characterized by alterations in key pathways such as tryptophan metabolism and fatty acid metabolism, with propionic and gluconic acids identified as central metabolic nodes; these changes are significantly associated with maternal testosterone levels and are differentially modulated by maternal obesity.

**WHAT IS KNOWN ALREADY:**

PCOS is known to adversely affect maternal and fetal metabolic health, carrying risks for intergenerational metabolic programming. However, the precise molecular nature of metabolic and lipidomic dysregulation at the maternal–fetal interface, particularly the specific drivers of hyperandrogenism and its complex interplay with maternal obesity, remains poorly characterized.

**STUDY DESIGN, SIZE, DURATION:**

This prospective comparative study evaluated 48 pregnant women with PCOS and 50 healthy pregnant controls. All biological samples were collected at the time of term delivery.

**PARTICIPANTS/MATERIALS, SETTING, METHODS:**

Participants were recruited from a tertiary care center. To minimize labor-induced metabolic confounding, all subjects underwent elective term cesarean sections. Untargeted metabolomics and lipidomics analyses were performed on strictly standardized maternal-side placental tissue and umbilical cord serum using liquid chromatography–mass spectrometry. Statistical analyses included multivariable linear regression to adjust for confounders and LASSO regression for key feature selection. Participants were further stratified by pre-pregnancy BMI (obese: BMI ≥28 kg/m^2^ vs non-obese: BMI <28 kg/m^2^) to assess the impact of maternal adiposity.

**MAIN RESULTS AND THE ROLE OF CHANCE:**

PCOS pregnancies showed significantly distinct metabolomic and lipidomic profiles compared to controls in both placental tissue (407 differential metabolites, 186 differential lipids; FDR < 0.05) and umbilical cord serum (330 differential metabolites, 314 differential lipids; FDR < 0.05). Key enriched KEGG pathways included tryptophan metabolism, sphingolipid metabolism, and biosynthesis of unsaturated fatty acids. Maternal testosterone levels demonstrated strong and independent correlations with several key differential metabolites and lipids. Propionic acid and gluconic acid were identified as central metabolic nodes. Subgroup analysis revealed that maternal obesity exerts an additive metabolic burden that partially masks the intrinsic PCOS molecular phenotype. The role of chance was minimized by appropriate sample size, statistical adjustment for confounders, and the use of FDR correction for multiple comparisons where applicable.

**LARGE SCALE DATA:**

N/A.

**LIMITATIONS, REASONS FOR CAUTION:**

The cross-sectional design and single-center setting limit causal and longitudinal inferences. Methodological constraints, including the absence of pre-pregnancy hormonal profiles, the lack of concurrent maternal peripheral blood sampling, and the restriction to maternal-side placental tissue and elective cesarean deliveries, necessitate caution when extending these findings to systemic maternal metabolism or vaginal births.

**WIDER IMPLICATIONS OF THE FINDINGS:**

These findings significantly advance our understanding of the metabolic pathophysiology of PCOS during pregnancy, highlighting specific pathways and molecules at the maternal–fetal interface. This knowledge could pave the way for identifying novel biomarkers for early risk stratification and developing targeted interventions to mitigate adverse metabolic programming in offspring of mothers with PCOS, potentially tailored by maternal obesity status.

**STUDY FUNDING/COMPETING INTEREST(S):**

This study was supported by the Sichuan Science and Technology Program (2026NSFSC1657), the ‘Talent Program’ Cultivation Project of Chengdu Women’s and Children’s Central Hospital (No. YC2023002), the Health Commission of Sichuan Province (No. 20PJ184), and the Medical Scientific Research Project Funded by the Chengdu Municipal Health Commission (No. 2024247). The authors declare no competing interests.

WHAT DOES THIS MEAN FOR PATIENTS? Polycystic ovary syndrome (PCOS) is a common endocrine disorder that can increase the risk of pregnancy complications and affect the long-term health of the baby. However, exactly how PCOS changes the ‘chemical environment’ inside the uterus where the baby grows has remained a mystery.

In this study, we zoomed in on the maternal–fetal interface, which serves as the crucial meeting point between mother and baby. We analyzed thousands of tiny molecules (metabolites and lipids) in the placenta and the baby’s umbilical cord blood immediately after birth in mothers with and without PCOS. We discovered that PCOS creates a highly distinct chemical environment during pregnancy. Specifically, the placenta in PCOS mothers showed a dramatic reduction in essential structural lipids. We also found that higher levels of maternal testosterone, a hallmark of PCOS, strongly drove these changes, throwing key energy and protective molecules out of balance. Interestingly, if a mother with PCOS also had obesity, her weight added an extra layer of metabolic stress to the placenta and baby.

Our findings act as a high-resolution ‘molecular map’ of a PCOS pregnancy. By understanding exactly which chemicals are disrupted by high testosterone and obesity, doctors may eventually be able to design personalized diets or specific medical treatments to protect the intrauterine environment. This offers new hope for preventing long-term health issues in the children of mothers with PCOS.

## Introduction

PCOS is the most common endocrine disorder affecting women of reproductive age, with a global prevalence of 11–13% (Bozdag *et al.*, 2016; Teede *et al.*, 2023). It is characterized by hyperandrogenism, ovulatory dysfunction, and polycystic ovarian morphology, frequently accompanied by insulin resistance and obesity (Stener-Victorin *et al.*, 2024). Beyond its well-documented impact on fertility, PCOS is increasingly recognized as a complex systemic metabolic disorder with profound long-term health implications for both mothers and their offspring (Balen *et al.*, 2016).

Pregnancy represents a critical window of metabolic adaptation, during which the maternal–fetal interface, primarily the placenta, acts as a dynamic hub orchestrating nutrient transfer and metabolic signaling essential for fetal development (Bowman *et al.*, 2021). In women with PCOS, these physiological adaptations may be compromised by pre-existing metabolic dysregulation, including hyperandrogenism, insulin resistance, and dyslipidemia (Stener-Victorin *et al.*, 2024). Consequently, PCOS pregnancies are associated with elevated risks of adverse obstetric outcomes, such as gestational diabetes mellitus, preeclampsia, and preterm birth (Mills *et al.*, 2020; Bahri Khomami *et al.*, 2024). Moreover, growing evidence suggests that maternal PCOS may program metabolic dysfunction in offspring through fetal exposure to an adverse intrauterine environment (Dumesic *et al.*, 2014; Vanky *et al.*, 2019; Risal *et al.*, 2023).

Obesity is a common comorbidity in PCOS, affecting ∼50% of patients (Lim *et al.*, 2012; Glueck and Goldenberg, 2019). The interplay between PCOS and obesity represents a critical area of investigation, as obesity may exacerbate PCOS-associated disturbances and independently impair placental function and fetal development (Lim *et al.*, 2013). However, few studies have systematically examined how obesity modifies the metabolic landscape of PCOS pregnancies, leaving it unclear whether identified alterations are PCOS-specific, obesity-driven, or the result of their synergistic interaction.

Untargeted metabolomics and lipidomics provide powerful platforms for profiling small-molecule metabolites and lipids, offering systemic insights into cellular metabolism and signaling (Wang *et al.*, 2020). In the context of PCOS, analyzing placental tissue and umbilical cord blood can reveal specific metabolic pathway perturbations that contribute to adverse programming. While previous research has identified metabolic disturbances in non-pregnant women with PCOS (Chang *et al.*, 2017; Sun *et al.*, 2019; Qian *et al.*, 2024), the placental–fetal metabolic landscape remains largely unexplored, particularly the relationship between maternal hyperandrogenism and these intricate molecular shifts.

The present study aimed to characterize the metabolomic and lipidomic profiles of placental tissue and umbilical cord serum in PCOS pregnancies compared to healthy controls. By integrating clinical data with obesity stratification, we sought to elucidate the distinct metabolic signatures of PCOS with and without obesity. Our findings provide comprehensive insights into the metabolic reprogramming associated with PCOS during pregnancy, potentially informing personalized intervention strategies to mitigate adverse intergenerational outcomes.

## Materials and methods

### Ethics statement

This prospective study adhered to the Declaration of Helsinki and the International Conference on Harmonisation Good Clinical Practice E6 (ICH-GCP) guidelines. The protocol was approved by the Research Ethics Committee of the Chengdu Women’s and Children’s Central Hospital (Approval No: 202096). All participants provided written informed consent prior to enrollment.

### Study design and population

This study recruited pregnant women diagnosed with PCOS and healthy controls from Chengdu Women’s and Children’s Central Hospital between January 2020 and December 2021. The final cohort comprised 48 women with PCOS and 50 healthy controls. PCOS was diagnosed according to the 2003 Rotterdam criteria, requiring at least two of the following: oligo- or anovulation, clinical or biochemical hyperandrogenism, and polycystic ovarian morphology on ultrasound, after excluding other etiologies (Rotterdam ESHRE ASRM-Sponsored PCOS Consensus Workshop Group, 2004). The control group consisted of age-matched pregnant women with regular menstrual cycles and no clinical signs of hyperandrogenism. Exclusion criteria for both groups included: (i) hypertensive disorders of pregnancy; (ii) gestational diabetes mellitus; (iii) intrahepatic cholestasis of pregnancy; (iv) placenta accreta spectrum disorders; and (v) other severe maternal metabolic, cardiovascular, or immune-related diseases. To minimize the potential confounding effects of labor stress and vaginal delivery on placental and fetal metabolism, all enrolled participants underwent elective cesarean section.

### Clinical data collection and definitions

Maternal demographic and anthropometric data were collected from electronic medical records. Pre-pregnancy BMI was calculated at the first antenatal visit during the first trimester (8–10 gestational weeks), which served as a proxy for pre-pregnancy status. Maternal weight was monitored throughout pregnancy, and the final weight was recorded upon admission for delivery. Gestational weight gain (GWG) was defined as the difference between the weight at delivery and the weight at the first antenatal visit. Maternal venous blood samples were collected at admission prior to cesarean section for the measurement of serum testosterone and other biochemical parameters. Neonatal outcomes, including gender, birth weight, and Apgar scores, were recorded immediately after birth.

### Sample collection

Placental tissue and umbilical cord blood samples were collected immediately after delivery. Placental tissue (1 cm × 1 cm × 1 cm) was excised from the central region of the maternal side, carefully avoiding macroscopic vessels, calcification, and infarcted areas. The tissue was rinsed thoroughly with 0.9% saline to remove residual blood, blotted dry, and immediately snap-frozen in liquid nitrogen. Umbilical cord blood was collected and centrifuged at 1690 *g* for 10 min at 4 °C to isolate serum. Samples showing visible hemolysis were excluded from the study. All specimens were stored at −80°C until metabolomic and lipidomic analyses.

### Biochemical analysis

Umbilical cord serum biochemical parameters, including triglycerides (TG), total cholesterol (TC), high-density lipoprotein cholesterol (HDL-C), low-density lipoprotein cholesterol (LDL-C), glucose (GLU), and glycated serum protein (GSP), were measured using commercial kits (Leidu, Shenzhen, China; Changchun Huili, Changchun, China) on an automatic biochemical analyzer following standardized clinical laboratory protocols. Maternal serum testosterone levels (Cloud-Clone Corp, Katy, TX, USA) and umbilical cord serum insulin levels (Multi Sciences, Hangzhou, China) were quantified using ELISA kits, strictly adhering to the manufacturers’ instructions.

### Metabolomics and lipidomics analyses

#### Sample preparations

All samples were processed according to standardized protocols (Zheng *et al.*, 2023; Ge *et al.*, 2025). For metabolomics, placental tissue (30 mg) was homogenized at 25 Hz for 5 min with 20 μl of internal standards (2-chloro-L-phenylalanine and fexofenadine), 100 μl of water, and 600 μl of methanol/acetonitrile (1:1, v/v). The mixture was centrifuged at 13 400 *g* for 10 min at 4 °C using an Eppendorf Centrifuge 5427R (Eppendorf, Hamburg, Germany). The supernatant was collected and evaporated to dryness using a Labconco CentriVap vacuum concentrator (Labconco, Kansas City, MO, USA) at 40 °C. The dried residue was reconstituted in 100 μl of 50% aqueous acetonitrile (v/v) for analysis. For umbilical cord serum metabolomics, 100 μl of serum was mixed with 20 μl of internal standards and 600 μl of methanol to precipitate proteins, followed by centrifugation, drying, and reconstitution in 100 μl of 50% aqueous acetonitrile. For lipidomics, lipids were extracted using the methyl tert-butyl ether (MTBE) method (Matyash *et al.*, 2008). Placental tissue (30 mg) or umbilical cord serum (100 μl) was mixed with 20 μl of internal standards, 100 μl of methanol, and 500 μl of MTBE. The mixture was homogenized (tissue) or vortexed (serum), sonicated, and phase-separated by adding water. The upper organic phase containing lipids was collected, dried, and reconstituted in 100 μl of methanol/dichloromethane (1:1, v/v).

#### Liquid chromatography–mass spectrometry analysis

Liquid chromatography–mass spectrometry (LC–MS) analysis was performed using a Waters ACQUITY UPLC I-Class system coupled to a Xevo G2-XS QTOF mass spectrometer (Waters, Milford, MA, USA). Chromatographic separation was achieved on an ACQUITY UPLC HSS T3 column (1.8 μm, 2.1 mm × 100 mm) equipped with a VanGuard pre-column, maintained at 40 °C. The injection volume was 5 μl. For metabolomics, the mobile phases consisted of 0.1% formic acid in water (Phase A) and acetonitrile (Phase B) for positive mode, or 5 mM ammonium formate in water (Phase A) and acetonitrile (Phase B) for negative mode. The flow rate was 0.4 ml/min with a gradient elution from 2% to 99% B. For lipidomics, the mobile phases were 60:40 acetonitrile/water with 10 mM ammonium formate (Phase A) and 90:10 isopropanol/acetonitrile with 10 mM ammonium formate (Phase B). The flow rate was 0.2 ml/min with a gradient elution from 30% to 100% B.

Mass spectrometry data were acquired in MSE continuum mode, which simultaneously collects low-energy (precursor ion) and high-energy (fragment ion) spectra. The electrospray ionization (ESI) source parameters were: capillary voltage 3.0 kV, sampling cone 30 V, source temperature 120 °C, and desolvation gas flow of 800 l/h. The mass range was set to 50–1200 m/z.

#### Data preprocessing

Raw LC–MS data were imported into Progenesis QI (version 3.0; Waters) for peak alignment, peak picking, and deconvolution. Positive and negative ion modes were processed separately. To ensure data integrity, only samples with complete data acquisition in both ionization modes were retained for downstream analysis. Features with a relative standard deviation >30% in pooled quality control (QC) samples were removed to ensure analytical reliability. The filtered data were imported into R (version 4.4.1; Vienna, Austria) using the tidyMass package (Shen *et al.*, 2022). Missing values were imputed using the k-nearest neighbors algorithm. To correct for inter-sample variability, data were normalized by probabilistic quotient normalization. Metabolites and lipids were putatively annotated by matching accurate mass *(m/z)* and retention time against public databases, including HMDB and LIPID MAPS. Lipids were classified according to the LIPID MAPS system, with classes defined by polar head groups and species differentiated by carbon chain saturation or length (Liebisch *et al.*, 2013).

### Statistical analysis

Clinical data are presented as mean ± SD or median (interquartile range [IQR]) for continuous variables. Categorical variables are expressed as frequencies and percentages. Differences between two groups were assessed using the Student’s *t*-test or Mann–Whitney *U*-test for continuous variables, and the chi-square test or Fisher’s exact test for categorical variables. For comparisons among four subgroups, one-way ANOVA or the Kruskal–Wallis test was applied. A two-sided *P*-value < 0.05 was considered statistically significant. All statistical analyses were conducted using R (version 4.4.1; Vienna, Austria).

Prior to multivariate and differential analysis, metabolite and lipid intensities were log-transformed and auto-scaled to satisfy the assumptions of normality and homogeneity of variance. Differential abundance analysis was performed using the limma R package (Ritchie *et al.*, 2015). Multivariable linear regression models adjusted for potential confounders: maternal age, pre-pregnancy BMI, gestational age, and GWG were employed. The Partial Least Squares Discriminant Analysis (PLS-DA) models were validated using 7-fold cross-validation and permutation tests for visualizing metabolic alterations. Differential features were identified based on false discovery rate (FDR) and variable importance in projection (VIP), with FDR < 0.05 and VIP > 1. To minimize multicollinearity among co-regulated metabolites and identify a robust set of key features with high predictive value, Least Absolute Shrinkage and Selection Operator (LASSO) regression was applied. The optimal penalty parameter (lambda) was determined by 5-fold cross-validation, and model stability was evaluated by bootstrap resampling. Pathway enrichment analysis was performed using the KEGG database via the clusterProfiler R package (Xu *et al.*, 2024), and network analysis was visualized using MetaboAnalyst 6.0 (Pang *et al.*, 2024).

To assess whether maternal obesity modifies PCOS-associated metabolic changes, a PCOS-by-BMI interaction term was included in the multivariable linear regression models. To further validate this interaction, subgroup analyses were conducted by stratifying participants based on the Chinese obesity criterion (BMI ≥ 28 kg/m^2^) into four groups: non-obese control, obese control, non-obese PCOS, and obese PCOS (Zhou, 2002).

## Results

### Baseline characteristics of the study population

**Figure 1.**
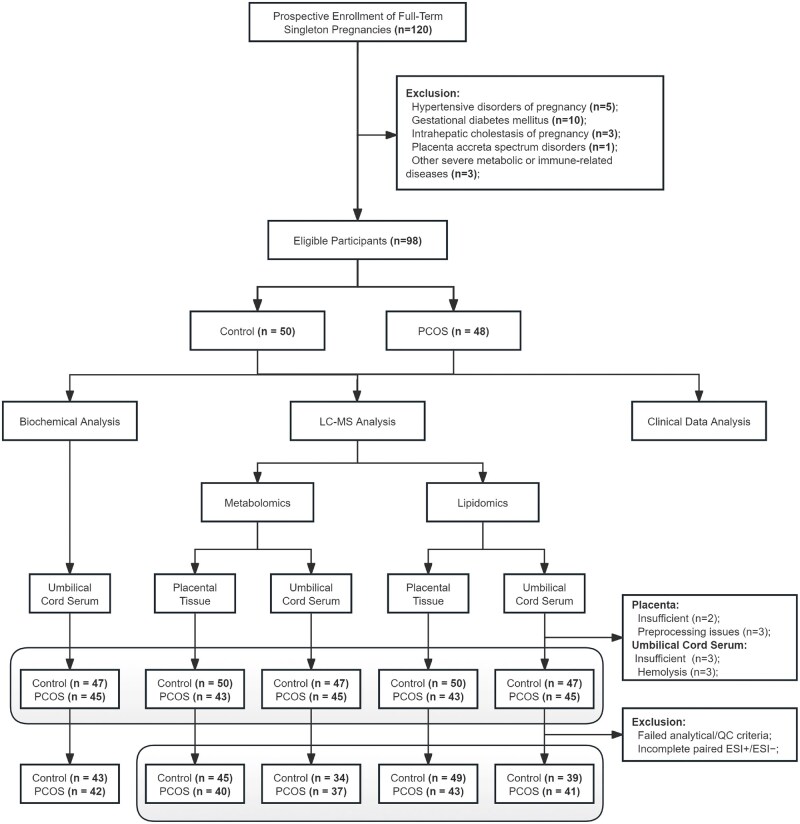
**Research flowchart**. PCOS, polycystic ovary syndrome; LC–MS, liquid chromatography–mass spectrometry; ESI+/−, positive and negative electrospray ionization modes.

The study included 50 control and 48 PCOS pregnant women (Fig. 1). Compared to controls, the PCOS group exhibited significantly higher maternal BMI [median (IQR): 27.89 (26.28–31.73) vs 27.27 (25.42–28.95), *P *= 0.04] and testosterone levels [median (IQR): 2648.54 (2104.25–3033.45) vs 1319.21 (1123.53–1491.93), *P *< 0.001]. In umbilical cord serum, levels of TG and GSP were significantly lower in the PCOS group compared to controls [TG: 0.24 (0.19–0.28) vs 0.26 (0.23–0.30), *P *= 0.012; GSP: 0.85 ± 0.17 vs 0.99 ± 0.1, *P *< 0.001]. No significant differences were observed in maternal age, gestational age, GWG, neonatal gender, birth weight, Apgar scores, or TSH (*P *> 0.05). Similarly, other lipid and glucose metabolism parameters showed no statistically significant differences between groups (*P *> 0.05) (Table 1).

**Table 1.** Clinical characteristics of PCOS and control groups.

	Control	PCOS	Statistic	*P*-value
	(n = 50)	(n = 48)		
**Age (years)**	27 (25–30.75)	28 (25–31)	1200	1.000
**BMI (kg/m^2^)**	27.27 (25.42–28.95)	27.89 (26.28–31.73)	910.5	**0.040** *
**Gestational age (weeks)**	39 (38–39)	39 (38–39)	1264	0.629
**GWG (kg)**	13.5 (11.12–17)	13.5 (10–15.25)	1421	0.116
**Neonatal gender (%)**			–	0.843
Female	25 (50)	25 (52.08)		
Male	25 (50)	23 (47.92)		
**Birthweight (g)**	3160 (2980–3327.5)	3140 (2950–3460)	1166	0.812
**Apgar score**				
1 min	10 (10–10)	10 (10–10)	1266	0.489
5 min	10 (10–10)	10 (10–10)	1225	0.317
10 min	10 (10–10)	10 (10–10)	1225	0.317
**TSH (mIU/l)**	1.41 (0.92–2.15)	1.57 (1.1–2.04)	1094	0.967
**Testosterone (pg/ml)**	1319.21 (1123.53–1491.93)	2648.54 (2104.25–3033.45)	74	**<0.001** *
**TG (mmol/l)**	0.26 (0.23–0.3)	0.24 (0.19–0.28)	1189.5	**0.012** *
**TC (mmol/l)**	1.44 (1.25–1.62)	1.36 (1.15–1.62)	1011	0.345
**HDL-C (mmol/l)**	0.8 (0.67–0.93)	0.76 (0.65–0.84)	1009.5	0.351
**LDL-C (mmol/l)**	0.48 (0.39–0.55)	0.48 (0.4–0.61)	843.5	0.604
**GLU (mmol/l)**	3.04 ± 0.66	2.89 ± 0.97	0.86	0.391
**GSP (mmol/l)**	0.99 ± 0.1	0.85 ± 0.17	1359	**<0.001** *
**Insulin (pmol/l)**	15.95 (8.12–22.5)	11.05 (5.09–30.14)	853	0.6

Continuous data are presented as median (IQR), categorical data as n (%). Continuous variables using *t*-test or Mann–Whitney *U*-test, categorical variables were compared using chi-square or Fisher’s exact test.

* *P *< 0.05 indicates statistical significance. GWG, gestational weight gain; TSH, thyroid-stimulating hormone; TG, triglycerides; TC, total cholesterol; HDL-C, high-density lipoprotein cholesterol; LDL-C, low-density lipoprotein cholesterol; GLU, glucose; GSP, glycated serum protein.

### Differential metabolic profiles in placenta tissue and umbilical cord serum

**Figure 2.**
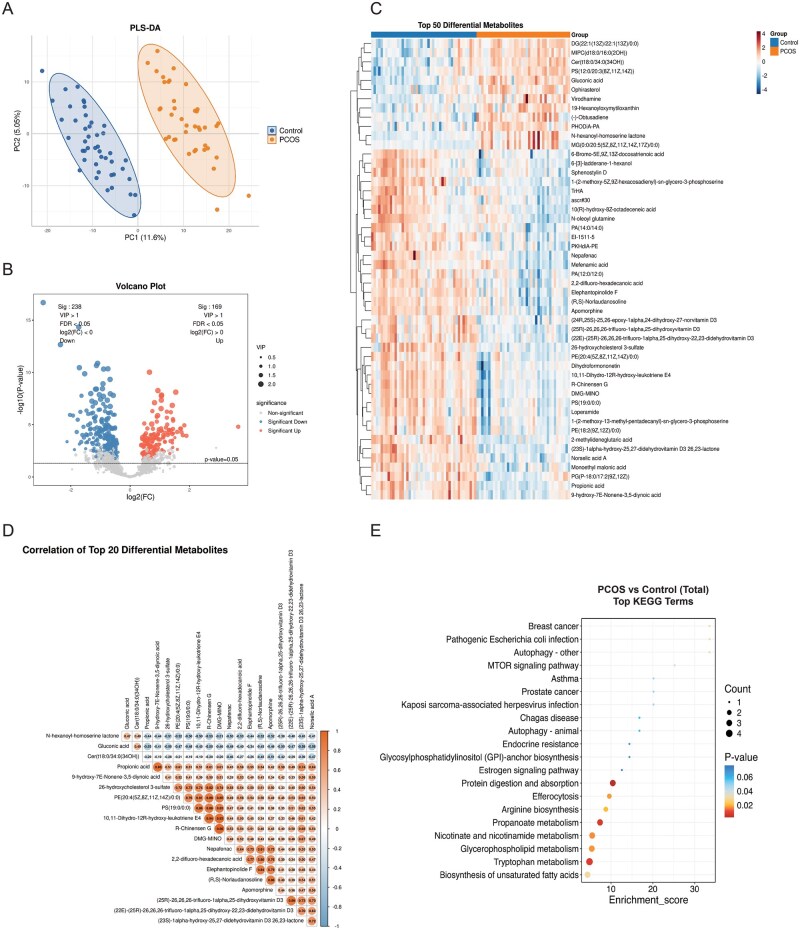
**Metabolomics analysis of placental tissue**. (**A**) PLS-DA score plot demonstrating metabolic separation between the PCOS and control groups; (**B**) Volcano plot of differential metabolites adjusted for confounders via multivariable linear regression (VIP > 1, FDR < 0.05; red: upregulated, blue: downregulated); (**C**) Heatmap of the top 50 differential metabolites (red: higher abundance, blue: lower abundance); (**D**) Correlation heatmap of the top 20 differential metabolites (orange: positive correlation, blue: negative correlation); (**E**) KEGG pathway enrichment bubble plot for the top 20 differential metabolites. PLS-DA, partial least squares discriminant analysis; PCOS, polycystic ovary syndrome; VIP, variable importance in projection; FDR, false discovery rate; KEGG, Kyoto Encyclopedia of Genes and Genomes.

**Figure 3.**
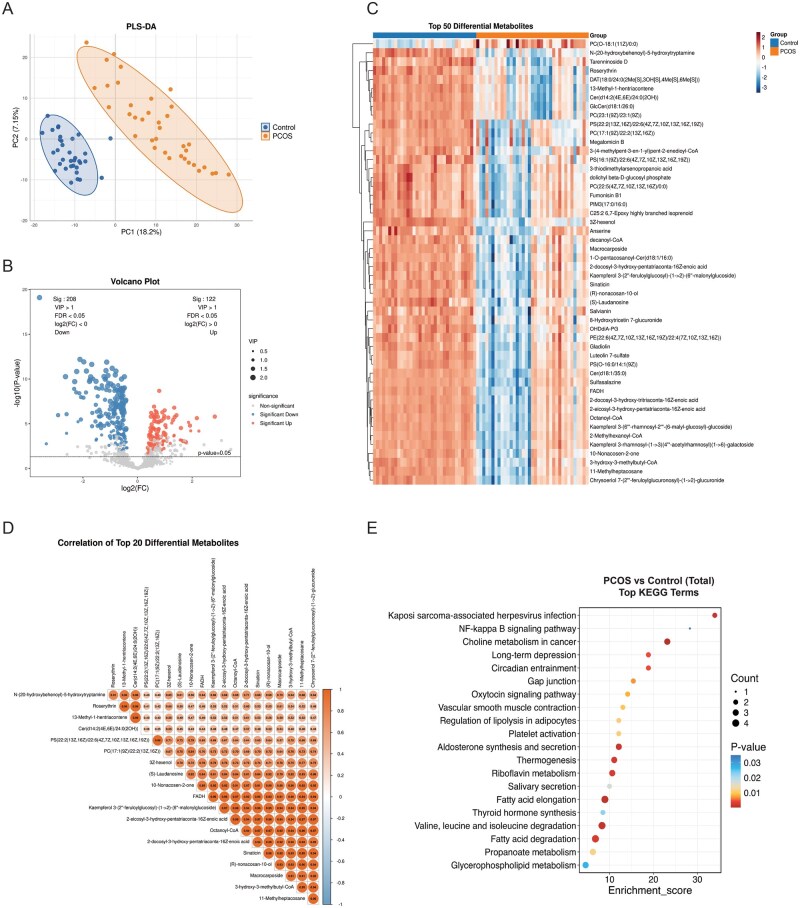
**Metabolomics analysis of umbilical cord serum**. (**A**) PLS-DA score plot demonstrating metabolic separation between the PCOS and control groups; (**B**) Volcano plot of differential metabolites adjusted for confounders (VIP > 1, FDR < 0.05; red: upregulated, blue: downregulated); (**C**) Heatmap of the top 50 differential metabolites (red: higher abundance, blue: lower abundance); (**D**) Correlation heatmap of the top 20 differential metabolites (orange: positive correlation, blue: negative correlation); (**E**) KEGG pathway enrichment bubble plot for the top 20 differential metabolites. PLS-DA, partial least squares discriminant analysis; PCOS, polycystic ovary syndrome; VIP, variable importance in projection; FDR, false discovery rate; KEGG, Kyoto Encyclopedia of Genes and Genomes.

Untargeted metabolomics analysis was performed on placental tissue and umbilical cord serum samples. Due to objective factors such as hemolysis or pre-processing issues, five placental and six umbilical cord serum samples were excluded prior to LC–MS analysis. Following strict matching of positive and negative ionization modes (ESI+/−) and QC, 85 placental tissue samples and 71 umbilical cord serum samples were ultimately retained for downstream analysis (Fig. 1). PLS-DA score plots revealed distinct metabolic separation between the PCOS and control groups in both tissue types (Figs 2A and 3A). To account for baseline phenotypic differences, multivariable linear regression was applied to adjust for maternal age, pre-pregnancy BMI, gestational age, and GWG (unadjusted results are presented in Supplementary Fig. S1A–D).

In placental tissue, 407 differential metabolites were identified after adjustment (FDR < 0.05, VIP > 1). These included 169 upregulated metabolites (e.g. gluconic acid, ophirasterol) and 238 downregulated metabolites (e.g. propionic acid, monoethyl malonic acid) in the PCOS group compared to controls (Fig. 2B and C; Supplementary File S1). Correlation analysis indicated strong co-regulation patterns among these differential metabolites (Fig. 2D). KEGG pathway analysis revealed significant enrichment in tryptophan metabolism, propanoate metabolism, and glycerophospholipid metabolism, as well as cellular processes such as efferocytosis and autophagy (Fig. 2E).

In umbilical cord serum, 330 differential metabolites were identified, comprising 122 upregulated and 208 downregulated metabolites (Fig. 3B, Supplementary File S1). Hierarchical clustering analysis of the top 50 differential metabolites revealed a striking pattern: while samples in the control group exhibited a relatively uniform high-abundance profile, the PCOS group displayed considerable heterogeneity, with a subset of samples showing distinct downregulation patterns (Fig. 3C). Correlation analysis confirmed significant associations among differential metabolites (Fig. 3D). KEGG pathway analysis highlighted pathways including fatty acid elongation; valine, leucine, and isoleucine degradation; and propanoate metabolism, alongside cellular processes such as autophagy (Fig. 3E).

### Differential lipidomic profiles in placenta tissue and umbilical cord serum

**Figure 4.**
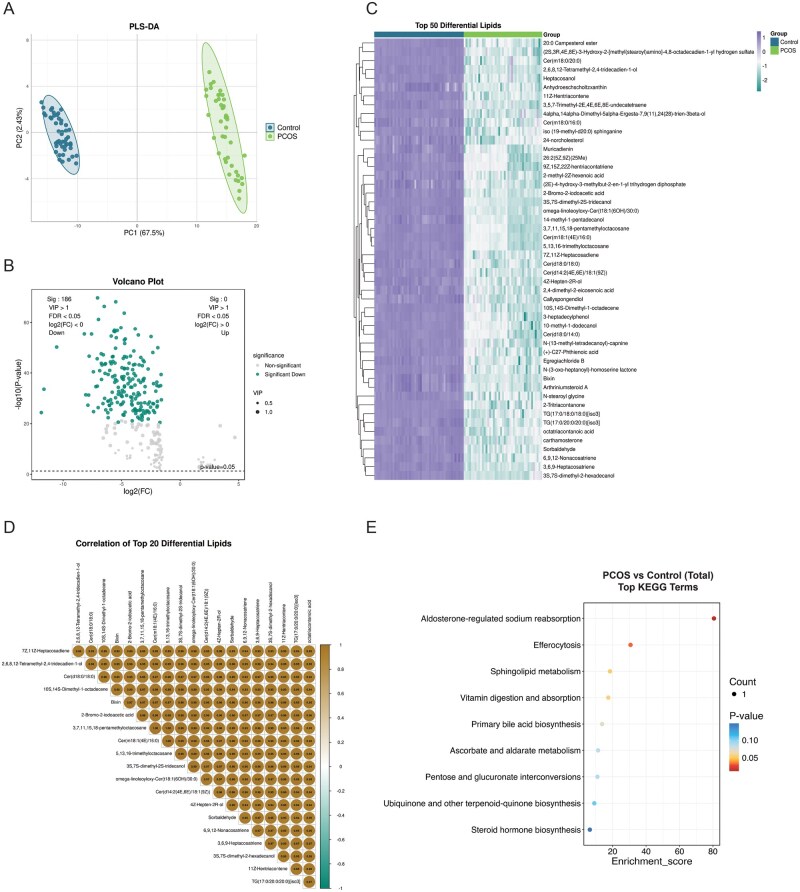
**Lipidomics analysis of placental tissue**. (**A**) PLS-DA score plot showing lipidomic separation between PCOS and control groups; (**B**) Volcano plot of differential lipids after adjustment for confounders (VIP > 1, FDR < 0.05; purple: upregulated, green: downregulated); (**C**) Heatmap of top 50 differential lipids (purple: upregulated, green: downregulated); (**D**) Correlation heatmap of top 20 differential lipids (gold: positive, green: negative); (**E**) KEGG enrichment bubble plot for top 20 differential lipids. PLS-DA, partial least squares discriminant analysis; PCOS, polycystic ovary syndrome; VIP, variable importance in projection; FDR, false discovery rate; KEGG, Kyoto Encyclopedia of Genes and Genomes.

**Figure 5.**
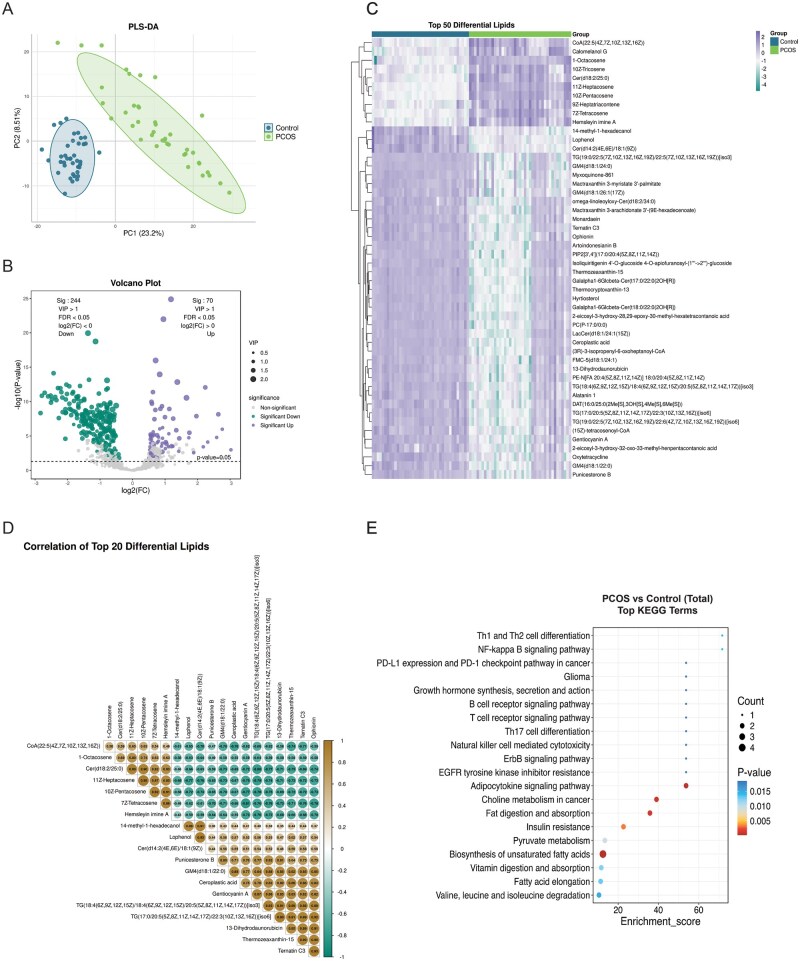
**Lipidomics analysis of umbilical cord serum**. (**A**) PLS-DA score plot showing lipidomic separation between PCOS and control groups; (**B**) Volcano plot of differential lipids after adjustment for confounders (VIP > 1, FDR < 0.05; purple: upregulated, green: downregulated); (**C**) Heatmap of top 50 differential lipids (purple: upregulated, green: downregulated); (**D**) Correlation heatmap of top 20 differential lipids (gold: positive, green: negative); (**E**) KEGG enrichment bubble plot for top 20 differential lipids. PLS-DA, partial least squares discriminant analysis; PCOS, polycystic ovary syndrome; VIP, variable importance in projection; FDR, false discovery rate; KEGG, Kyoto Encyclopedia of Genes and Genomes.

Following strict matching of positive and negative ionization modes (ESI+/−) and QC, 92 placental tissue samples and 80 umbilical cord serum samples were retained for lipidomics analysis (Fig. 1). PLS-DA demonstrated clear separation of lipidomic profiles between PCOS and control groups in both matrices (Figs 4A and 5A). Similar to the metabolomics analysis, confounder adjustment was performed (unadjusted results in Supplementary Fig. S2A–D).

Lipid composition analysis indicated that placental lipids were primarily composed of fatty acids and conjugates (FA), hydrocarbons (HC), and fatty alcohols (FAl). Notably, the overall abundance of these classes was significantly lower in the PCOS group compared to controls (Supplementary Fig. S3A, Supplementary File S2). In placental tissue, 186 differential lipids were identified post-adjustment, all of which were downregulated in the PCOS group, including octatriacontanoic acid and carboceric acid (Fig. 4B and C; Supplementary File S1). Strong correlations were observed among these lipids (Fig. 4D). KEGG pathway analysis revealed enrichment in sphingolipid metabolism and efferocytosis-related pathways (Fig. 4E).

In umbilical cord serum, lipid composition included flavonoids (Fla), FA, and isoprenoids (IP), with no significant differences in total class abundance between groups (Supplementary Fig. S3B, Supplementary File S2). Differential analysis identified 314 lipids, with 70 upregulated (e.g. CoA(22:5(4Z, 7Z, 10Z, 13Z, 16Z)), calomelanol G) and 244 downregulated (e.g. 14-methyl-1-hexadecanol, lophenol) (Fig. 5B and C; Supplementary File S1). Consistent with the metabolomic findings, hierarchical clustering of lipids revealed greater heterogeneity within the PCOS group compared to the more uniform profile observed in controls (Fig. 5C). Correlation analysis confirmed significant associations among these differential lipids (Fig. 5D). KEGG pathway analysis highlighted lipid metabolism pathways (biosynthesis of unsaturated fatty acids, fatty acid elongation) and organismal systems pathways (adipocytokine signaling, fat digestion, and absorption), as well as efferocytosis-related pathways (Fig. 5E).

### Identification of key differential metabolites and lipids

LASSO regression with bootstrap validation was employed to select key differential features from the identified pool. In placental tissue metabolomics, 25 key metabolites were selected, comprising 12 metabolites positively associated with PCOS and 13 negatively associated (Supplementary Fig. S4A–C). In umbilical cord serum metabolomics, 10 key metabolites were identified, with 1 positively and 9 negatively associated with PCOS (Supplementary Fig. S5A–C). For placental tissue lipidomics, 15 key lipids were selected, all of which were negatively associated with PCOS (Supplementary Fig. S6A–C). In umbilical cord serum lipidomics, 10 key lipids were identified, including 6 positively and 4 negatively associated with PCOS (Supplementary Fig. S7A–C).

### Correlation between key metabolites and clinical variables

Spearman correlation analysis was performed to evaluate the associations between these key molecular features and maternal clinical variables (BMI, testosterone, TG, and GSP). Significant correlations were defined as *P *< 0.05, with |r| ≥ 0.3 indicating moderate associations and |r| ≥ 0.5 denoting strong associations.

**Figure 6.**
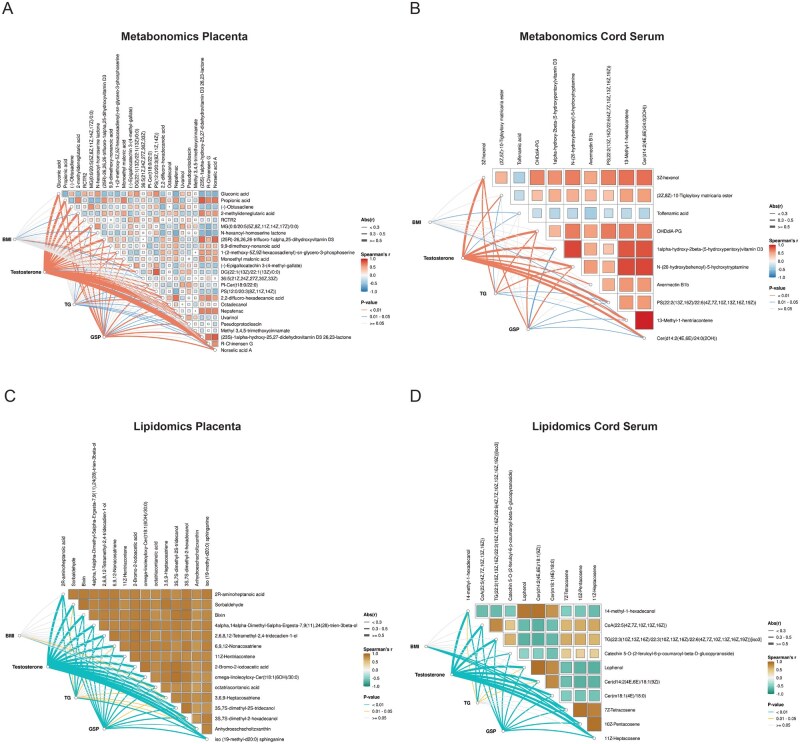
**Correlation analysis of clinical variables with key differential metabolites and lipids**. (**A, B**) Network heatmaps illustrating correlations between clinical variables and key differential metabolites from (A) placental tissue and (B) umbilical cord serum; (**C, D**) Network heatmaps illustrating correlations between clinical variables and key differential lipids from (C) placental tissue and (D) umbilical cord serum.

In placental tissue metabolomics, testosterone exhibited significant correlations with 23 key metabolites. Notably, gluconic acid showed a strong positive correlation (*r* = 0.57), while propionic acid displayed a strong negative correlation (*r* = −0.65). GSP correlated with 10 metabolites, including a moderate positive correlation with 2-methylideneglutaric acid (*r* = 0.44) and a negative correlation with gluconic acid (*r* = −0.44). TG showed only one moderate correlation, and BMI showed none (Fig. 6A). In umbilical cord serum metabolomics, testosterone was negatively correlated with nine metabolites, with OHDdiA-PG (*r* = −0.69) and 3Z-hexenol (*r* = −0.67) showing strong negative associations. GSP correlated with six metabolites, including a moderate positive correlation with 3Z-hexenol (*r* = 0.41). TG correlated with three metabolites, while BMI showed no significant correlations (Fig. 6B).

In placental tissue lipidomics, testosterone and GSP were significantly correlated with all 15 key lipids. Testosterone showed strong negative correlations with octatriacontanoic acid (*r* = −0.75) and 3,6,9-heptacosatriene (*r* = −0.74), while GSP showed a moderate positive correlation with 6,9,12-nonacosatriene (*r* = 0.47). TG correlated with three lipids, and BMI showed none (Fig. 6C). In umbilical cord serum lipidomics, testosterone correlated with 10 lipids, showing a strong positive correlation with TG(22:3/22:3/22:6)[iso3] (*r* = 0.71) and a strong negative correlation with Cer(d14:2/18:1) (*r* = −0.71). GSP correlated with 10 lipids, including a moderate positive correlation with Cer(m18:1/18:0) (*r* = 0.44). BMI and TG each showed moderate correlations with three lipids (Fig. 6D). Detailed correlation data are provided in Supplementary File S3.

To further quantify the contribution of testosterone to metabolic variations, linear regression analysis was performed. In placental tissue metabolomics, norselic acid A (*R*^2^ = 0.38, *P *< 0.001) and propionic acid (*R*^2^ = 0.35, *P *< 0.001) showed the strongest linear dependence on testosterone levels. In umbilical cord serum metabolomics, 3Z-hexenol (*R*^2^ = 0.44, *P *< 0.001) and OHDdiA-PG (*R*^2^ = 0.34, *P *< 0.001) exhibited significant linear relationships (Supplementary Figs S8 and S9). For placental lipidomics, all key lipids showed strong linear correlations with testosterone, with 3S, 7S-dimethyl-2-hexadecanol, 6,9,12-nonacosatriene, and 2-bromo-2-iodoacetic acid explaining the highest variance (*R*^2^ = 0.58, *P *< 0.001) (Supplementary Fig. S10). Similarly, umbilical cord serum lipids, such as 7Z-tetracosene (*R*^2^ = 0.51, *P *< 0.001) and 10Z-pentacosene (*R*^2^ = 0.51, *P *< 0.001), were strongly predicted by testosterone levels (Supplementary Fig. S11). In contrast, BMI, TG, and GSP showed weak linear relationships with these key features (*R*^2^ < 0.3) (Supplementary Figs S12, S13, S14, S15, S16, S17, S18, S19, S20, S21, S22, and S23).

### Integrative analysis of key differential metabolites and pathways

**Figure 7.**
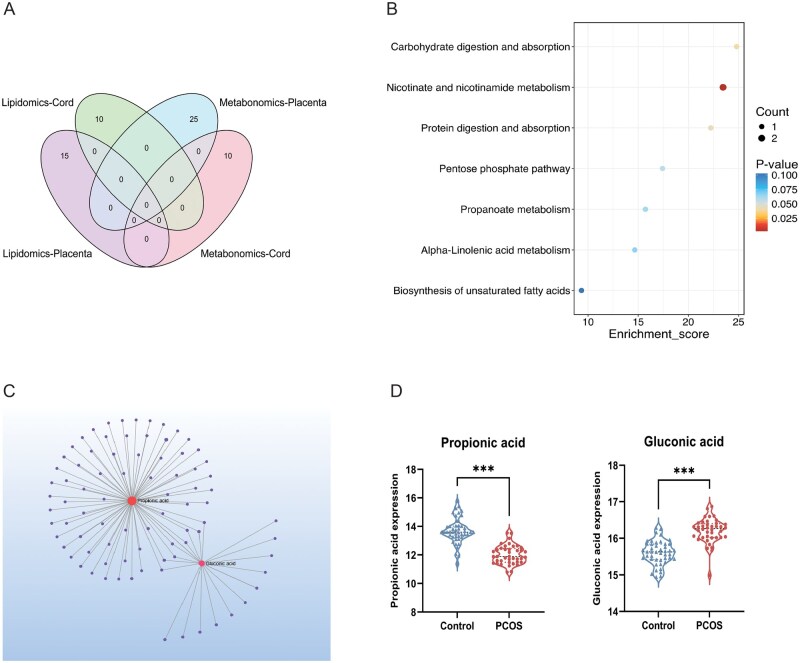
**Identification of core metabolic nodes and pathways across multi-omics datasets**. (**A**) Venn diagram illustrating the overlap of differential features across placental tissue and umbilical cord serum; (**B**) KEGG enrichment bubble plot for core differential metabolites; (**C**) Network analysis depicting biochemical interactions among key metabolic nodes; (**D**) Violin plots showing the distribution and expression levels of central metabolites in PCOS and control groups. KEGG, Kyoto Encyclopedia of Genes and Genomes; PCOS, polycystic ovary syndrome; Statistical significance was assessed by an unpaired Student’s *t*-test. ***P < 0.001 compared to the control group.

Venn diagram analysis revealed limited overlap between differential metabolites and lipids across placental tissue and umbilical cord serum (Fig. 7A), suggesting tissue-specific metabolic reprogramming in PCOS pregnancies. KEGG enrichment analysis of the combined key features identified significant pathways, including nicotinate and nicotinamide metabolism, carbohydrate digestion and absorption, and protein digestion and absorption (Fig. 7B). Network analysis further positioned propionic acid and gluconic acid as central hub nodes connecting the metabolic and lipidomic alterations (Fig. 7C, Supplementary File S4). Consistent with this, the expression profiles of these key node metabolites showed distinct and significant differences between the PCOS and control groups (Fig. 7D).

### Interaction of PCOS and obesity on differential metabolite and lipid profiles

Given the high prevalence of obesity in PCOS, the combined effects of PCOS and obesity were systematically evaluated. Interaction analyses were first performed on clinical phenotypes, and effect sizes were visualized in Supplementary Fig. S24. Testosterone levels were consistently elevated in women with PCOS regardless of BMI status, with no significant interaction observed (*P *> 0.05). Similarly, for umbilical cord serum biochemical parameters, while distinct trends were noted in TC and LDL-C, no statistically significant interactions between PCOS and obesity were detected (*P *> 0.05). Subsequently, subgroup analysis was performed based on BMI (≥28 kg/m^2^), dividing participants into four subgroups: 33 non-obese controls, 17 obese controls, 26 non-obese PCOS cases, and 22 obese PCOS cases (Table 2). Subgroup comparisons revealed significant differences in TG (*P *= 0.036) and GSP (*P *< 0.001) across the four groups, beyond the expected differences in testosterone and BMI. Specifically, post hoc analysis indicated that TG levels were significantly elevated in the obese control group compared to the non-obese PCOS group (*P *= 0.018). Notably, GSP levels were significantly lower in the non-obese PCOS group compared to both non-obese (*P *< 0.001) and obese controls (*P *= 0.001).

**Table 2.** Clinical characteristics of PCOS and control subgroups after stratification.

	Control		PCOS		
	Non-obese	Obese	Non-obese	Obese	*P*-value
	(n = 33)	(n = 17)	(n = 26)	(n = 22)	
**Age (years)**	28 (25–31)	25 (25–30)	27.5 (25–31)	29.5 (24.25–31)	0.557
**BMI (kg/m^2^)**	26.04 (24.44–27.2)	29.79 (28.98–30.86)	26.48 (24.82–27.21)	32.13 (30.2–33.41)	**<0.001** *
**Gestational age (weeks)**	39 (38–39)	39 (38–39)	39 (38–39)	38 (37–39)	0.061
**GWG (kg)**	14.08 ± 3.86	15.12 ± 4.07	12.63 ± 3.83	13.02 ± 8.34	0.299
**Neonatal gender (%)**					0.869
Female	16 (48.48)	9 (52.94)	15 (57.69)	10 (45.45)	
Male	17 (51.5)	8 (47.06)	11 (42.31)	12 (54.55)	
**Birthweight (g)**	3130 (2940–3320)	3290 (3100–3520)	3220 (3030–3480)	3130 (2920–3390)	0.647
**Apgar score**					
1 min	10 (10–10)	10 (10–10)	10 (10–10)	10 (9–10)	0.656
5 min	10 (10–10)	10 (10–10)	10 (10–10)	10 (10–10)	0.327
10 min	10 (10–10)	10 (10–10)	10 (10–10)	10 (10–10)	0.327
**TSH (mIU/l)**	1.34 (0.89–1.84)	1.44 (1.29–2.43)	1.57 (1.17–2.04)	1.51 (0.96–1.84)	0.7576
**Testosterone (pg/ml)**	1418.09 (1180.7–1498.9)	1229.3 (1088.34–1390.45)	2666.66 (2192.67–2996.1)	2531.54 (2081.61–3049.09)	**<0.001** *
**TG (mmol/l)**	0.25 (0.22–0.29)	0.27 (0.24–0.32)	0.21 (0.19–0.28)	0.24 (0.2–0.27)	**0.036** *
**TC (mmol/l)**	1.45 (1.32–1.62)	1.4 (1.15–1.62)	1.35 (1.11–1.6)	1.45 (1.2–1.64)	0.512
**HDL-C (mmol/l)**	0.82 ± 0.16	0.76 ± 0.19	0.76 ± 0.19	0.8 ± 0.24	0.523
**LDL-C (mmol/l)**	0.5 (0.39–0.52)	0.41 (0.38–0.6)	0.49 (0.41–0.56)	0.46 (0.37–0.62)	0.802
**GLU (mmol/l)**	2.99 ± 0.69	3.14 ± 0.62	2.9 ± 0.83	2.87 ± 1.18	0.786
**GSP (mmol/l)**	0.99 ± 0.11	0.99 ± 0.09	0.82 ± 0.17	0.91 ± 0.16	**<0.001** *
**Insulin (pmol/l)**	16.98 (8.14–21.9)	13.63 (9.3–24.81)	16.17 (6.93–32.97)	7.1 (3.67–18.5)	0.437

Continuous data are presented as median (IQR), categorical data as n (%). Group comparisons were performed using one-way ANOVA or the Kruskal–Wallis test for continuous variables, and the chi-square test or Fisher’s exact test for categorical variables, followed by Bonferroni or Dunn’s post hoc tests for pairwise comparisons, respectively.

* *P *< 0.05 indicates statistical significance. GWG, gestational weight gain; TSH, thyroid-stimulating hormone; TG, triglycerides; TC, total cholesterol; HDL-C, high-density lipoprotein cholesterol; LDL-C, low-density lipoprotein cholesterol; GLU, glucose; GSP, glycated serum protein.

**Figure 8.**
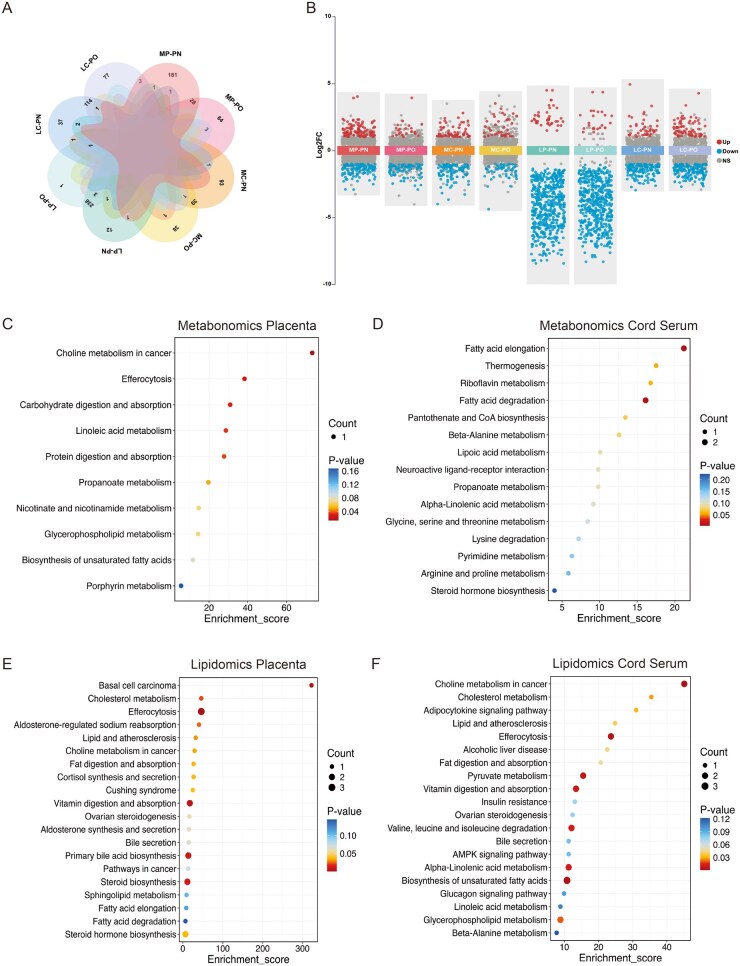
**Impact of obesity status on the PCOS-associated metabolic and lipidomic landscape**. (**A**) Venn diagrams showing significant features across stratification groups; (**B**) Grouped volcano plot illustrating significant metabolic alterations across four subgroups (|Log2 FC| > 1, FDR < 0.05; red: upregulated, blue: downregulated); (**C–F**) KEGG enrichment bubble plots of the top 20 differential features in obese PCOS pregnancies from: (C) placental metabolomics, (D) umbilical cord metabolomics, (E) placental lipidomics, and (F) umbilical cord lipidomics. Abbreviations for subgroups: MP-PN, Metabolomics-Placenta PCOS & Non-obese; MP-PO, Metabolomics-Placenta PCOS & Obese; MC-PN, Metabolomics-Cord serum PCOS & Non-obese; MC-PO, Metabolomics-Cord serum PCOS & Obese; LP-PN, Lipidomics-Placenta PCOS & Non-obese; LP-PO, Lipidomics-Placenta PCOS & Obese; LC-PN, Lipidomics-Cord serum PCOS & Non-obese; LC-PO, Lipidomics-Cord serum PCOS & Obese. PCOS, polycystic ovary syndrome; FC, fold change; FDR, false discovery rate; KEGG, Kyoto Encyclopedia of Genes and Genomes.

To characterize the molecular signatures modulated by the interaction between PCOS and obesity, a comprehensive interaction analysis was first performed on the omics data. Although there was no significant interaction effect for any individual metabolite or lipid for the PCOS-by-BMI interaction term (FDR < 0.05), stratified differential analyses revealed distinct metabolic patterns in obese and non-obese subgroups. For these subgroup comparisons (PCOS vs controls), significant differential features were defined as FDR < 0.05 and |log2 FC| > 1. In placental tissue, metabolomics analysis identified 224 differential metabolites in the non-obese subgroup and 102 in the obese subgroup; lipidomics analysis identified 284 and 270 differential lipids, respectively. In umbilical cord serum, metabolomics revealed 147 differential metabolites in the non-obese subgroup and 88 in the obese subgroup; lipidomics found 174 and 222 differential lipids, respectively (Fig. 8A and B).

To dissect biological functions specific to each BMI stratum, KEGG enrichment analysis was performed. In the non-obese PCOS subgroup, key enriched pathways included glycerophospholipid metabolism and propanoate metabolism in metabolomics, as well as fatty acid elongation and vitamin digestion and absorption in lipidomics (Supplementary Fig. S25A–F). Conversely, in the obese subgroup, differential metabolites were enriched in linoleic acid metabolism and propanoate metabolism in the placenta, and fatty acid elongation and degradation in the cord serum (Fig. 8C and D). Lipidomics analysis in the obese subgroup highlighted primary bile acid biosynthesis and steroid biosynthesis in the placenta, and biosynthesis of unsaturated fatty acids and pyruvate metabolism in the cord serum (Fig. 8E and F).

## Discussion

This study represents the first comprehensive metabolomic and lipidomic profiling of paired placental tissue and umbilical cord serum from pregnant women with PCOS. By integrating multi-omics approaches with clinical parameters and stratifying by obesity status, we identified distinct metabolic signatures associated with PCOS that may underlie the intergenerational transmission of metabolic dysfunction.

Clinical comparison confirmed that hyperandrogenism remains a hallmark of PCOS during pregnancy. Intriguingly, our umbilical cord serum analysis revealed significantly lower TG and GSP levels in the PCOS group compared to controls. This finding stands in contrast to some previous studies, which reported elevated lipids or glucose metabolism trends in PCOS offspring (Daan *et al.*, 2017; Sun *et al.*, 2020). However, our results are strongly supported by a gestationally hyperandrogenic nonhuman primate model of PCOS, which demonstrated significantly lower levels of free fatty acids in female fetuses during late gestation (Abbott *et al.*, 2010). This discrepancy highlights that the metabolic impact of maternal PCOS on the fetus is not universally adverse but rather modulated by a combination of clinical interventions and intrinsic placental adaptations. From a clinical perspective, the strict antenatal management and dietary control routinely implemented for PCOS pregnancies at our tertiary center likely mitigated fetal hyperglycemia and hyperlipidemia. Furthermore, our subgroup analysis dissected the roles of obesity and PCOS. We found that obesity was the primary driver for elevated TG levels (highest in the obese women without PCOS group), whereas PCOS status specifically was associated with lower GSP levels, even in non-obese women. This suggests a protective placental adaptation or altered nutrient transport in non-obese PCOS pregnancies, possibly driven by hyperandrogenism-mediated downregulation of glucose transporters, a mechanism that warrants further investigation. These findings underscore that the metabolic programming in PCOS offspring is not uniformly adverse but is highly modulated by maternal BMI and clinical management quality.

Metabolomic analysis suggests widespread metabolic dysregulation in the PCOS placenta and umbilical cord serum, affecting multiple pathways crucial for fetal development and placental function. Among these intricate metabolic networks, tryptophan (TRP) metabolism emerged as a profoundly affected cascade, particularly within the placental compartment. Specifically, we identified significant alterations in key TRP catabolites, including the downregulation of kynurenic acid (KYNA) and indoxyl, and the upregulation of indolepyruvate and 2-aminomuconic acid semialdehyde (ACMS). Approximately 95% of TRP is metabolized via the classical kynurenine pathway (KP) to generate kynurenine (KYN) and subsequently KYNA, ultimately leading to the biosynthesis of nicotinamide adenine dinucleotide (NAD^+^), while the remainder is metabolized into indole derivatives (Bender, 1983; Badawy, 2017). Previous studies have established that systemic KP activation (indicated by an increased KYN/TRP ratio) is closely associated with the pathogenesis of insulin resistance in metabolic disorders such as PCOS and obesity (Brandacher *et al.*, 2006; Oxenkrug, 2013; Jovanovic *et al.*, 2022). However, our findings reveal a distinct localized depletion of KYNA in the PCOS placenta. During pregnancy, KYN and its metabolites can cross the placenta and the fetal blood–brain barrier (Pedraz-Petrozzi *et al.*, 2023). KYNA has been shown to play an essential role in fetal growth, particularly in the development of the central nervous system *in utero* (Notarangelo and Schwarcz, 2016). Therefore, the significant reduction of KYNA in placental tissue observed in our study may compromise this protective microenvironment, potentially linking the maternal PCOS metabolic milieu to the increased risk of long-term neurodevelopmental disorders frequently reported in PCOS offspring. Furthermore, TRP metabolism is heavily co-regulated by the maternal gut microbiome (Jovanovic *et al.*, 2022). Metabolites such as indoxyl and indolepyruvate are classical microbial-derived indole derivatives. The significant shifts in these specific metabolites strongly point to maternal gut dysbiosis, a recognized pathological feature of PCOS, whose metabolic consequences cross the maternal–fetal interface and alter the placental metabolome (Li *et al.*, 2023).

Additionally, the downregulation of propionic acid and the upregulation of gluconic acid serve as central nodes of metabolic alteration in our network analysis. Propionic acid is a crucial short-chain fatty acid (SCFA) primarily produced by gut microbiota fermentation, playing pivotal roles in energy homeostasis, lipid metabolism, and immune regulation (He *et al.*, 2020; Ziętek *et al.*, 2021). Previous studies establish that propionic acid improves glucose homeostasis by inhibiting hepatic gluconeogenesis and regulates cholesterol metabolism by reducing fat storage (Macfarlane and Macfarlane, 2003). In the context of PCOS, the depletion of propionic acid at the maternal–fetal interface provides direct molecular evidence of maternal gut dysbiosis, a recognized hallmark of the syndrome. Crucially, our correlation analysis revealed a strong negative association between maternal testosterone and placental propionic acid levels. This supports a potential ‘androgen–microbiome–metabolite’ mechanistic axis, wherein maternal hyperandrogenism alters the gut microbiome composition, thereby suppressing the production of beneficial SCFAs. Recent *in vivo* evidence further corroborates this axis, demonstrating that supplementation with microbiome-derived propionic acid derivatives effectively ameliorates dehydroepiandrosterone (DHEA)-induced PCOS phenotypes through the aryl hydrocarbon receptor (AhR)-NOD-like receptor family pyrin domain-containing 3 (NLRP3) inflammasome pathway (Li *et al.*, 2025). Consequently, the localized deficit of propionic acid observed in our placental cohort may further exacerbate placental inflammation and insulin resistance. This depletion deprives the developing fetus of a critical metabolic and immunoregulatory substrate, ultimately contributing to adverse long-term fetal metabolic programming. Conversely, the upregulation of gluconic acid in PCOS placental tissue highlights profound alterations in local glucose utilization. While gluconic acid has been sporadically identified in previous metabolomic screens, its specific physiological implications in human pregnancy remain largely unexplored (Amereh *et al.*, 2025; Zarzoza-Mendoza *et al.*, 2025). Biochemically, gluconic acid is a direct product of the enzymatic or non-enzymatic oxidation of glucose, a reaction that concomitantly generates hydrogen peroxide (H_2_O_2_). Consequently, gluconic acid can serve as a robust indicator of oxidative stress. The increased gluconic acid levels observed in our cohort may reflect enhanced glucose oxidation or compromised antioxidant capacity in the PCOS placenta, which is perfectly consistent with previous reports of elevated oxidative stress in PCOS pregnancies (Boutzios *et al.*, 2013; Zhang *et al.*, 2019).

Lipidomic analysis revealed tissue-specific alterations in lipid profiles between PCOS and control pregnancies. In placental tissue, all identified differential lipids were remarkably downregulated in PCOS samples, indicating a widespread suppression of placental lipid synthesis or enhanced lipid catabolism. The predominant suppression of fatty acids and conjugates, HCs, and FAls is particularly significant given their critical functions in placental development. Fatty acids are essential precursors for prostaglandins and other eicosanoids that regulate vascular tone, inflammation, and parturition (Innes and Calder, 2018; Duttaroy and Basak, 2020). Their downregulation may compromise placental vascularization and perfusion, potentially contributing to the increased risk of placental insufficiency observed in PCOS pregnancies (Chavan-Gautam *et al.*, 2018; Kelley *et al.*, 2019b; Szczuko *et al.*, 2020). Moreover, HCs and FAls are important components of cellular membranes and participate in cell signaling pathways. Their reduction may impair placental barrier function and transcellular transport of nutrients and waste products between maternal and fetal circulations (Kelley *et al.*, 2019b). Crucially, our correlation analysis strongly linked these placental lipid alterations to maternal hyperandrogenism. Maternal testosterone exhibited robust negative correlations with placental lipids, particularly long-chain fatty acids. This suggests that testosterone may upregulate key lipid catabolic enzymes while downregulating lipid synthetic pathways, driving the global lipid depletion observed in the PCOS placenta.

In contrast to the uniform downregulation observed in the placenta, umbilical cord serum exhibited a bidirectional and more complex metabolic pattern. Specifically, countering the widespread fatty acid depletion observed in the placenta, we noted a distinct upregulation of highly unsaturated fatty acyl-CoAs in the fetal circulation. This indicates enhanced fatty acid activation for subsequent β-oxidation or incorporation into complex lipids, likely reflecting a compensatory fetal mechanism to adapt to the altered nutrient supply from the dysregulated placenta. Conversely, the downregulation of bioactive sterols suggests disrupted fetal sterol homeostasis, which has significant implications for neurodevelopment and metabolic programming, given the essential role of sterols in fetal brain myelination during late gestation (Woollett, 2005; Chatuphonprasert *et al.*, 2018). Furthermore, lipidomic analysis revealed relatively high levels of flavonoids in umbilical cord serum. Although there were no significant differences in total flavonoid abundance between PCOS and control pregnancies, their pronounced presence is most likely attributable to maternal dietary intake and efficient transplacental transfer, rather than disease-specific endogenous metabolic alterations. Prior pharmacokinetic and experimental studies consistently demonstrate that dietary flavonoids, such as quercetin, catechins, and other polyphenolic compounds, can cross the placenta and accumulate in fetal tissues, including the brain (Schroder-van Der Elst *et al.*, 1998; Chu *et al.*, 2006; Vanhees *et al.*, 2011). While flavonoids share this capacity to cross the placental barrier, different classes may exert distinct biological effects. For instance, quercetin and catechins exhibit potent antioxidant and anti-inflammatory activities, potentially mitigating oxidative stress, supporting trophoblast function, and promoting placental vascular remodeling (Gao *et al.*, 2018; Ebegboni *et al.*, 2019; Zhao *et al.*, 2024). Additionally, quercetin acts as an endocrine modulator, improving adiponectin signaling and placental structure in diabetic pregnancies (Mahabady *et al.*, 2021). However, a balanced perspective is necessary, as excessive *in utero* exposure to certain flavonoids has been linked to disrupted thyroid hormone transport and epigenetic reprogramming, carrying potential long-term metabolic consequences (van der Heide *et al.*, 2003; Vanhees *et al.*, 2011). Similar to the placenta, maternal testosterone levels were significantly correlated with specific cord serum lipids, indicating that the metabolic impact of maternal hyperandrogenism actively crosses the placental barrier to directly perturb fetal lipid homeostasis.

Collectively, the robust correlations observed between maternal testosterone levels and differential features across both the metabolomic and lipidomic compartments establish hyperandrogenism as a paramount driver of metabolic reprogramming in PCOS pregnancies. These findings align with previous studies demonstrating that androgens directly govern placental function and metabolism (Sun *et al.*, 2012; Koster *et al.*, 2015). The mechanistic connection between hyperandrogenism and this widespread dysregulation likely involves multiple intersecting pathways. Testosterone may directly alter key metabolic enzyme expression through genomic actions mediated by the androgen receptor, thereby regulating the transcription of target genes involved in lipid and glucose metabolism (Kelley *et al.*, 2019a). Additionally, androgens can exert non-genomic effects via membrane receptors, rapidly activating signaling cascades that further modulate these metabolic networks (Filippou and Homburg, 2017; Sanchez-Garrido and Tena-Sempere, 2020). Beyond these direct actions at the maternal–fetal interface, our findings highlight an indirect ‘androgen–microbiome–metabolite’ axis, wherein hyperandrogenism disrupts gut microbiota activity, further compounding the systemic and local metabolic dysfunction at the maternal–fetal interface. Furthermore, previous research has reported that a maternal hyperandrogenic environment during pregnancy may predispose offspring to fetal hyperandrogenism (Mehrabian and Kelishadi, 2012; Daan *et al.*, 2017; Homburg *et al.*, 2017; Barrett *et al.*, 2018). The complex relationships among the transgenerational transmission of androgens, placental–fetal metabolic reprogramming, and the long-term metabolic consequences for the offspring require further longitudinal investigation. Elucidating these mechanisms will be crucial for understanding the full scope of intergenerational metabolic programming in PCOS.

Beyond the intrinsic, androgen-driven metabolic reprogramming, maternal adiposity serves as a critical modifier of the maternal–fetal microenvironment in PCOS. Interestingly, our interaction analysis did not identify individual metabolites with statistically significant synergistic effects between these two conditions. This suggests that the metabolic impact of maternal obesity and PCOS on the placenta and fetus may be largely additive rather than synergistic, with each condition contributing independently to the adverse metabolic milieu. However, the distinct metabolic signatures observed in non-obese versus obese PCOS pregnancies further highlight the complex interplay between these two overlapping conditions. The intriguing finding that PCOS induces more extensive metabolic alterations in non-obese women suggests that the intrinsic, androgen-driven metabolic effects of PCOS may be partially masked or modified by the profound, systemic metabolic disturbances inherent to obesity itself. Furthermore, the enrichment of different metabolic pathways between non-obese and obese PCOS pregnancies implies potential mechanistic divergence based on maternal body composition. While glycerophospholipid and propanoate metabolism appear to represent core PCOS-specific metabolic signatures independent of adiposity, the specific enrichment of linoleic acid metabolism in obese PCOS women suggests that concomitant obesity may channel PCOS-related metabolic dysfunction through distinct pro-inflammatory pathways (Szczuko *et al.*, 2020). This differential metabolic reprogramming has important implications for developing targeted, phenotype-specific therapeutic, and nutritional interventions for different PCOS phenotypes during pregnancy.

Several limitations of this study should be acknowledged. First, the cross-sectional design at delivery provides a metabolic snapshot, precluding causal inferences and the capture of dynamic gestational changes. Second, while the overall sample size is substantial for omics research, sample attrition from strict QC limited the statistical power to detect subtle interactions in subgroup analyses. Third, although our standardized sampling strategically captures the maternal–fetal interface, the lack of concurrent maternal peripheral blood and fetal-side placental profiling precludes a truly systemic, spatially resolved metabolic overview. Fourth, although maternal testosterone was quantified at delivery, the lack of comprehensive pre-pregnancy baseline hormonal profiles prevents us from stratifying the cohort to further explore how specific PCOS sub-phenotypes might differentially drive the multi-omics alterations at the maternal–fetal interface. Finally, to standardize the metabolic baseline and avoid the profound confounding effects of labor stress, all deliveries occurred via elective cesarean section, which may limit generalizability to vaginal births. Future longitudinal studies with larger cohorts and matched maternal–placental–fetal sampling are warranted to validate these findings and explore phenotype-specific interventions.

## Conclusion

In conclusion, our comprehensive multi-omics profiling reveals profound, tissue-specific metabolic reprogramming at the maternal–fetal interface in PCOS pregnancies. We identified maternal hyperandrogenism as a paramount driver of these alterations, characterized by global metabolomic and lipidomic reprogramming and the dysregulation of central metabolic nodes, notably propionic and gluconic acids. Furthermore, our stratification demonstrates that maternal obesity exerts an additive metabolic burden that can mask the intrinsic PCOS metabolic phenotype. These findings provide a crucial molecular framework for understanding the intergenerational metabolic programming of PCOS. Ultimately, this knowledge highlights the urgent need to develop phenotype-specific, targeted therapeutic, and nutritional strategies to mitigate long-term adverse outcomes for both mothers with PCOS and their offspring.

## Supplementary Material

hoag036_Supplementary_Data

## Data Availability

The data presented in this study are available on request from the corresponding author.
