# Supplementary material for: Metabolic reprogramming in placenta and umbilical cord serum of polycystic ovary syndrome pregnancies: testosterone-associated alterations and interaction with obesity
Source: Hum Reprod Open. 2026 Apr 21;2026(3):hoag036. doi: 10.1093/hropen/hoag036 (PMC13186602; doi:10.1093/hropen/hoag036)
Supplement: hoag036_Supplementary_Data [file hoag036_supplementary_data.zip › Supplementary Information.docx]

**Supplementary Materials**

**Metabolic Reprogramming in Placenta and Umbilical Cord Serum of Polycystic Ovary Syndrome Pregnancies: Testosterone-Associated Alterations and Interaction with Obesity**

Huisheng Ge, Xiafei Wu, Dongni Huang, Li He, Dandan Liu, Lulu Wang, Liling Xiong, Dan Luo, Qiannan Hou, Hong Liu, Lunbo Tan, Yonghong Lin, Chang Chen, Xixi Wu


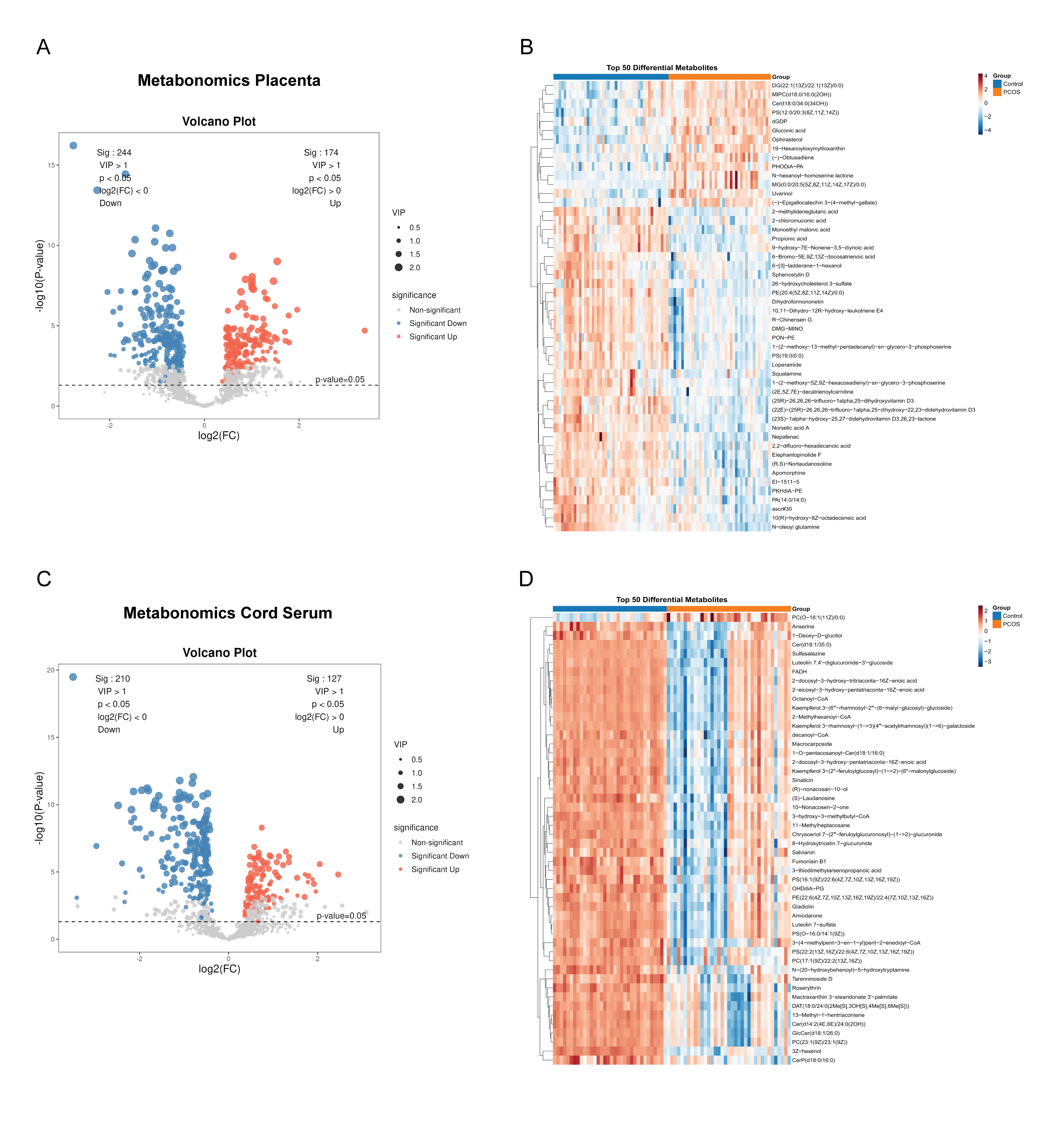


**Supplementary Figure S1.** **Metabolomics analysis before adjustment.** (A) Volcano plot of differential metabolites from placental tissue before multivariable adjustment (VIP > 1, *P*-value < 0.05); (B) Heatmap of the top 50 differential metabolites from placental tissue; (C) Volcano plot of differential metabolites from umbilical cord serum before adjustment; (D) Heatmap of the top 50 differential metabolites from umbilical cord serum.


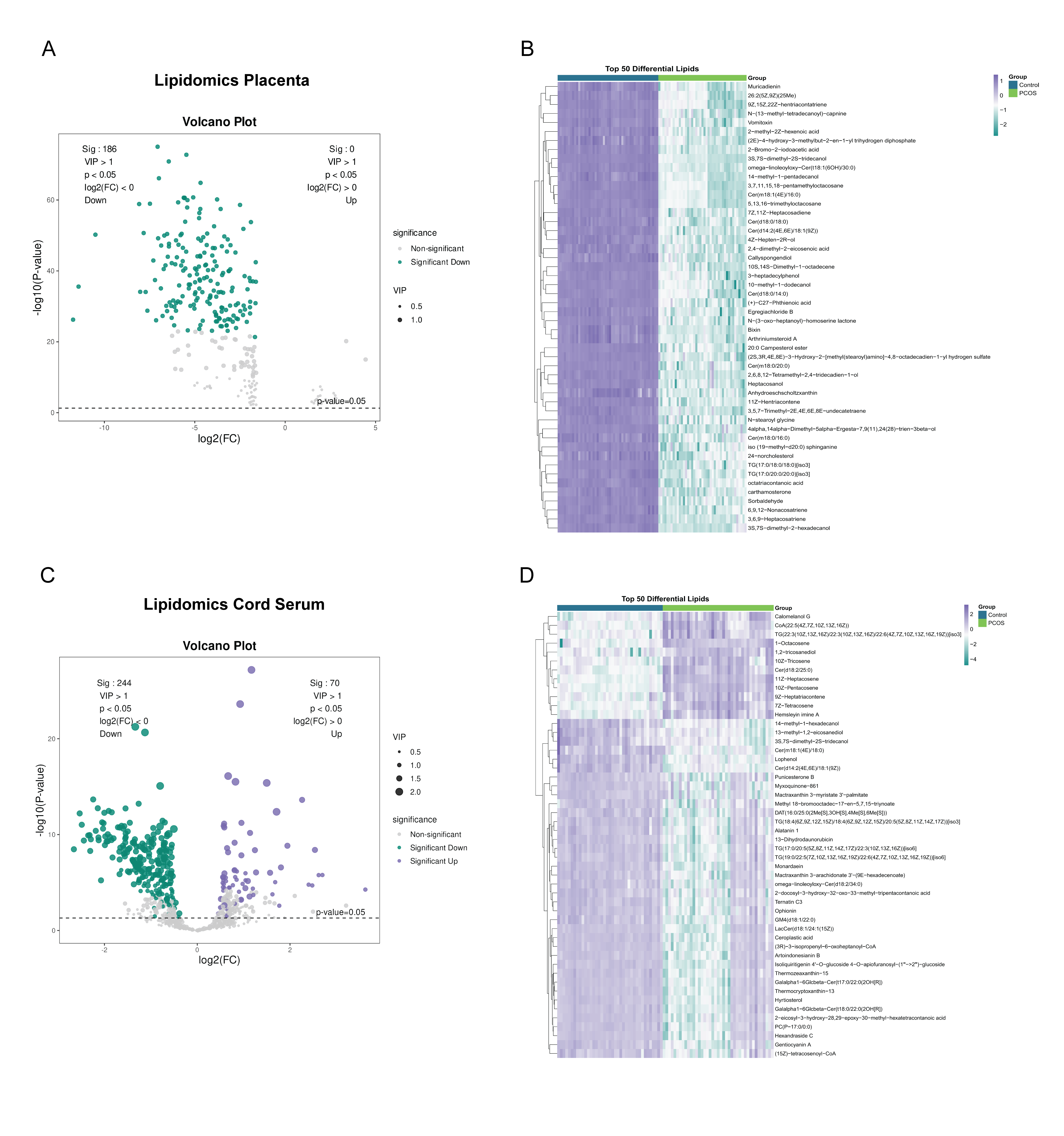


**Supplementary Figure S2.** **Lipidomics analysis before adjustment.** (A) Volcano plot of differential lipids from placental tissue before multivariable adjustment (VIP > 1, *P*-value < 0.05); (B) Heatmap of top 50 differential lipids from placental tissue; (C) Volcano plot of differential lipids from umbilical cord serum before adjustment; (D) Heatmap of top 50 differential lipids from umbilical cord serum.


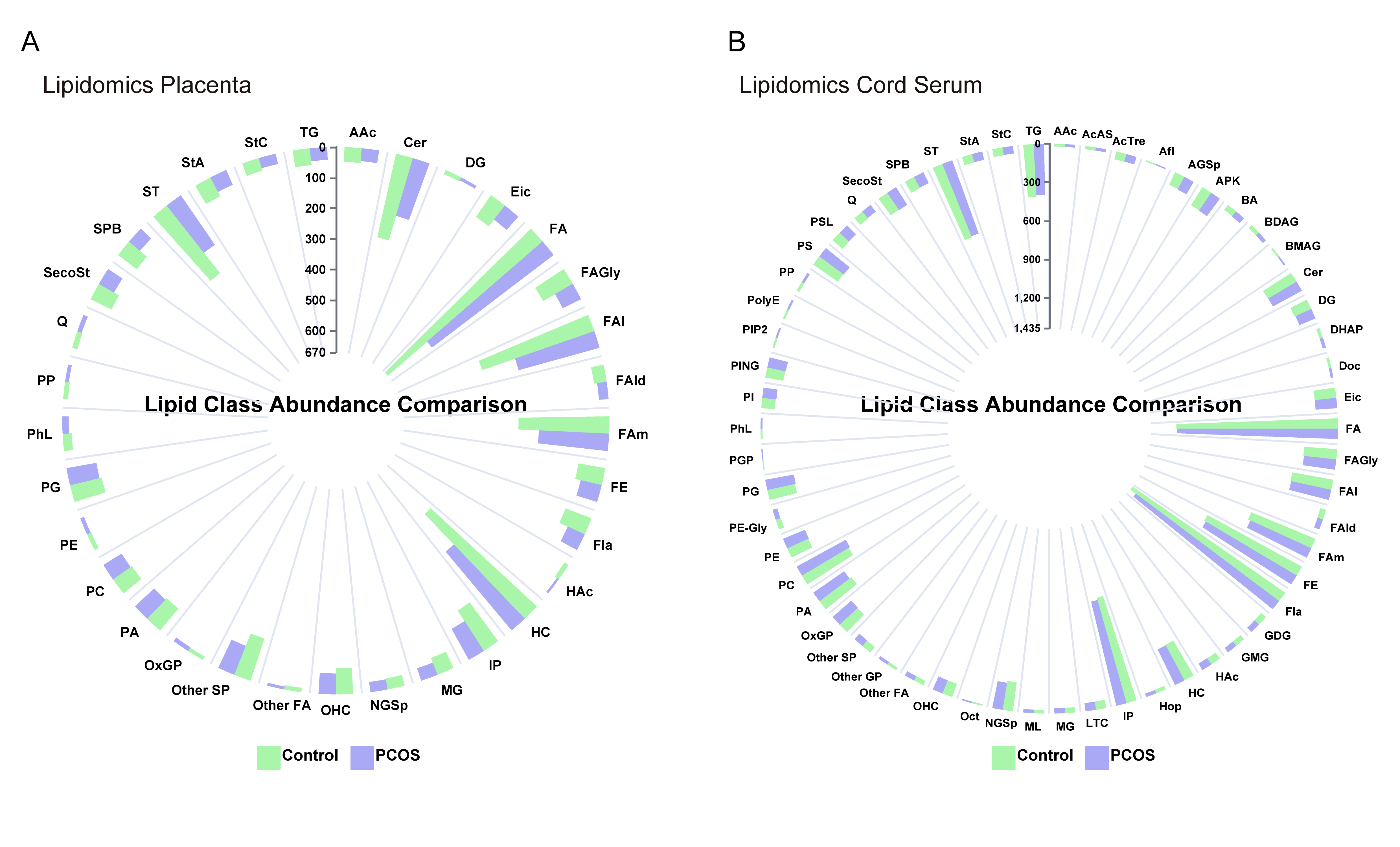


**Supplementary Figure S3.** **Lipid subclass composition analysis.** (A) Donut charts illustrating the lipid subclass composition in placental tissue for PCOS and control groups; (B) Donut charts illustrating the lipid subclass composition in umbilical cord serum for PCOS and control groups.


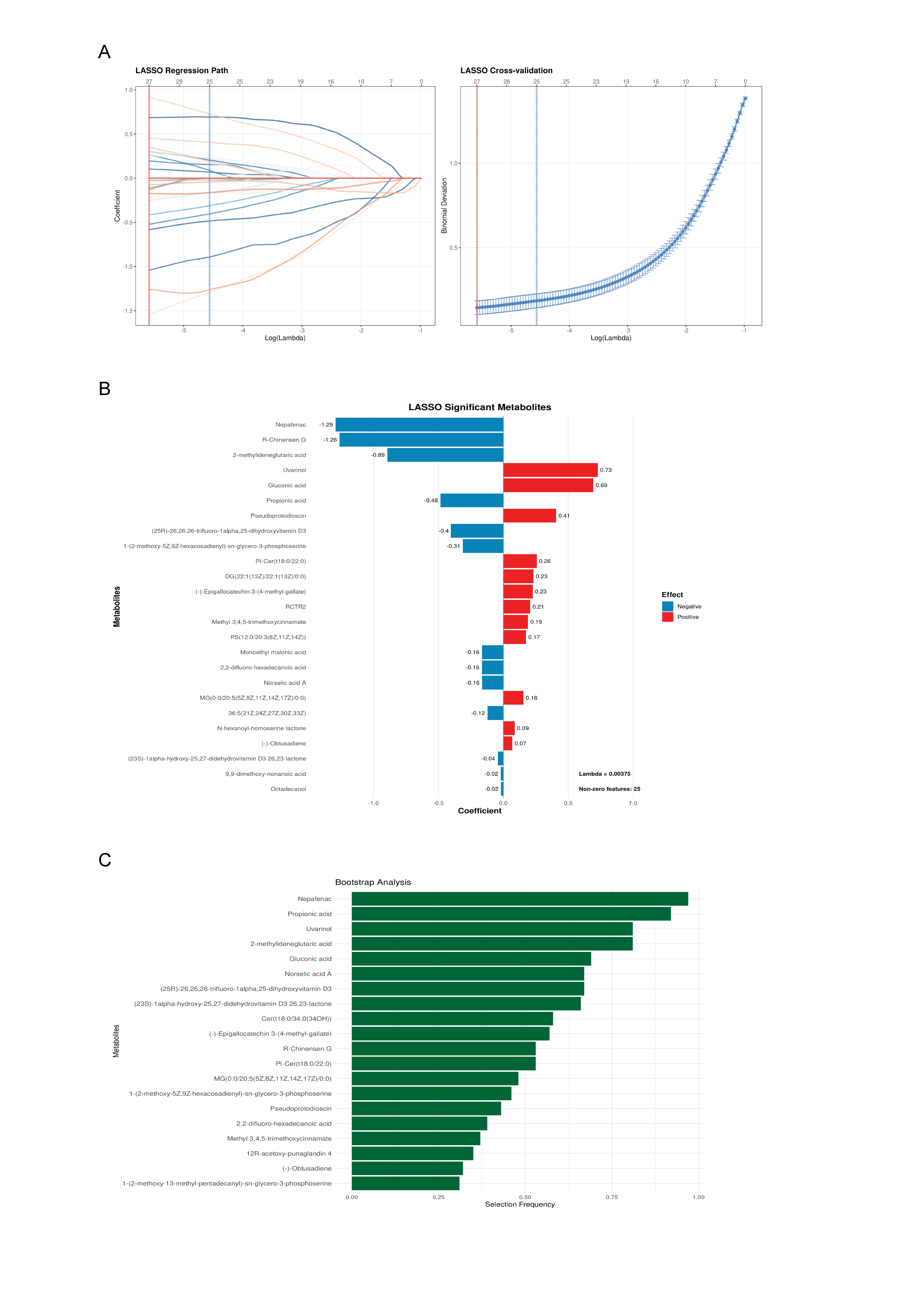


**Supplementary Figure S4.** **Selection of key differential metabolites from placental tissue.** (A) LASSO regression analysis, including the coefficient path plot and cross-validation plot. The red dashed line indicates lambda at minimum mean squared error (λ_min_); the blue dashed line indicates lambda at one standard error (λ_1se_); (B) Bar plot of key differential metabolites selected by LASSO regression with corresponding coefficients (red: positive association, blue: negative association); (C) Bar plot evaluating the stability of the top 20 differential metabolites using bootstrap analysis (n = 1000 iterations).


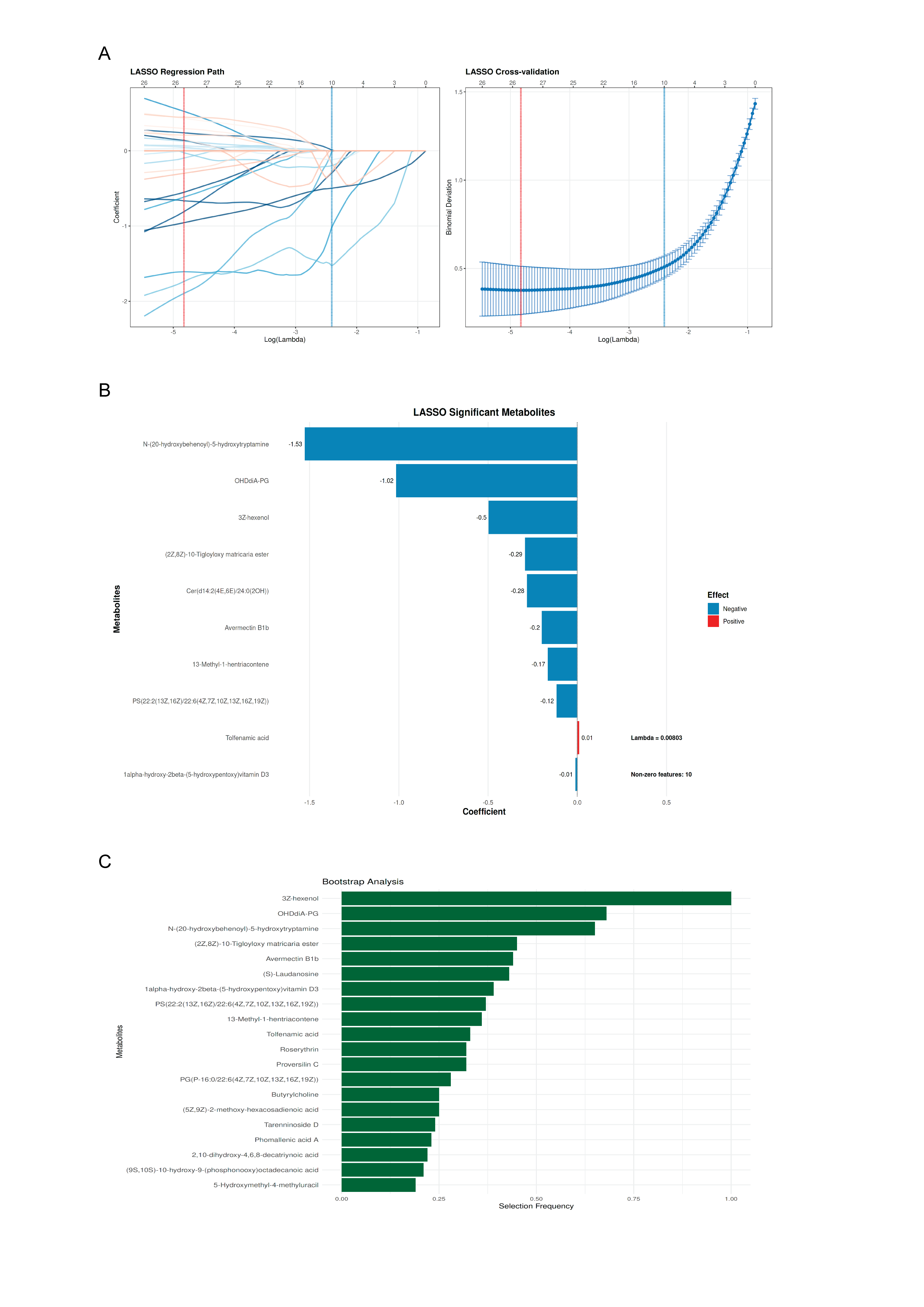


**Supplementary Figure S5. Selection of key differential metabolites from umbilical cord serum.** (A) LASSO regression analysis, including the coefficient path plot and cross-validation plot. The red dashed line indicates lambda at minimum mean squared error (λ_min_); the blue dashed line indicates lambda at one standard error (λ_1se_); (B) Bar plot of key differential metabolites selected by LASSO regression; (C) Stability evaluation of the top 20 differential metabolites via bootstrap analysis.


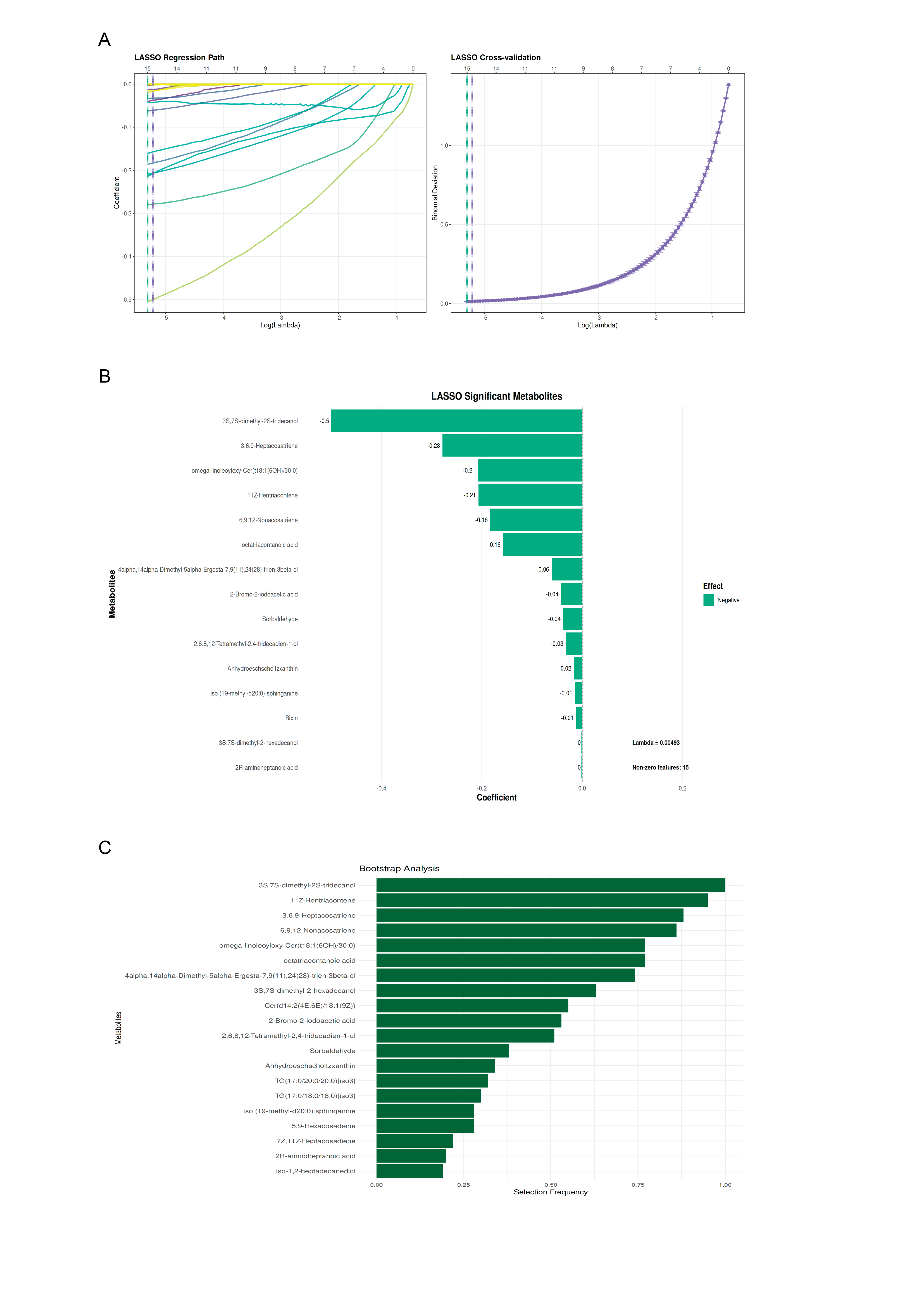


**Supplementary Figure S6.** **Selection of key differential lipids from placental tissue.** (A) LASSO regression analysis, including the coefficient path plot and cross-validation plot. The green dashed line indicates lambda at minimum mean squared error (λ_min_); the purple dashed line indicates lambda at one standard error (λ_1se_); (B) Bar plot of key differential lipids selected by LASSO regression (purple: positive association, green: negative association); (C) Bar plot evaluating the stability of the top 20 differential lipids using bootstrap analysis.


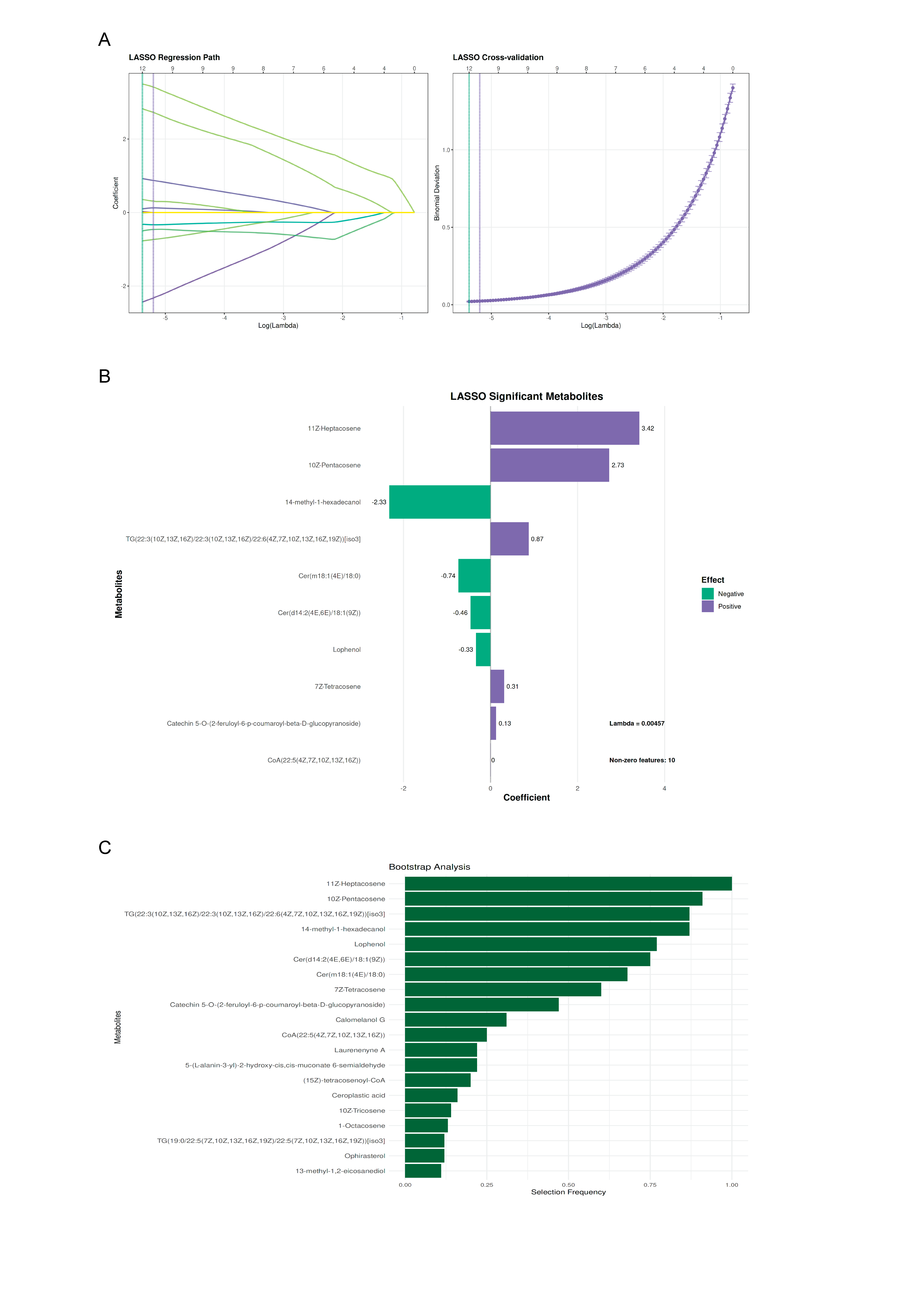


**Supplementary Figure S7. Selection of key differential lipids from umbilical cord serum.** (A) LASSO regression analysis, including the coefficient path plot and cross-validation plot. The green dashed line indicates lambda at minimum mean squared error (λ_min_); the purple dashed line indicates lambda at one standard error (λ_1se_); (B) Bar plot of key differential lipids selected by LASSO regression; (C) Stability evaluation of the top 20 differential lipids via bootstrap analysis.


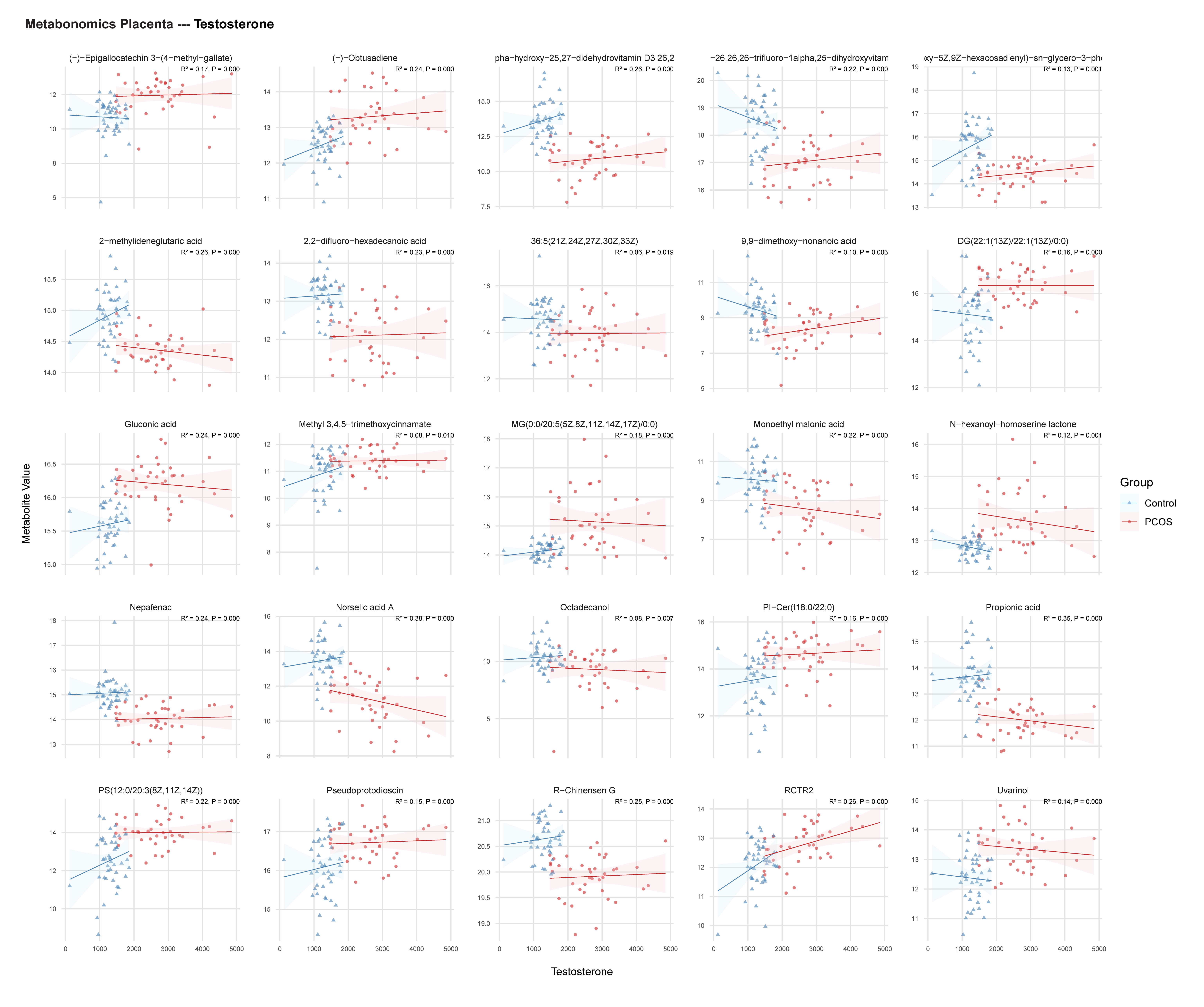


**Supplementary Figure S8.** Correlation analysis of key differential metabolites in metabolomics from placental tissue with maternal testosterone levels in PCOS and control groups.
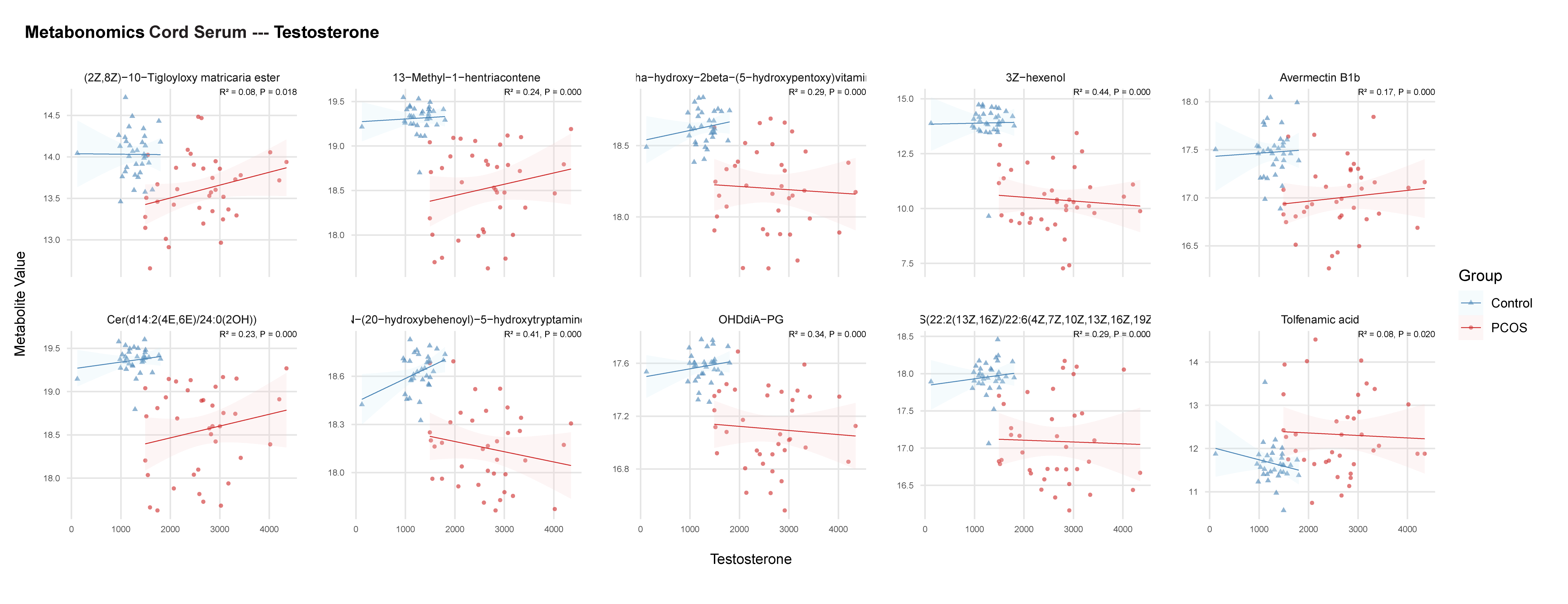


**Supplementary Figure S9.** Correlation analysis of key differential metabolites in metabolomics from umbilical cord serum with maternal testosterone levels in PCOS and control groups.


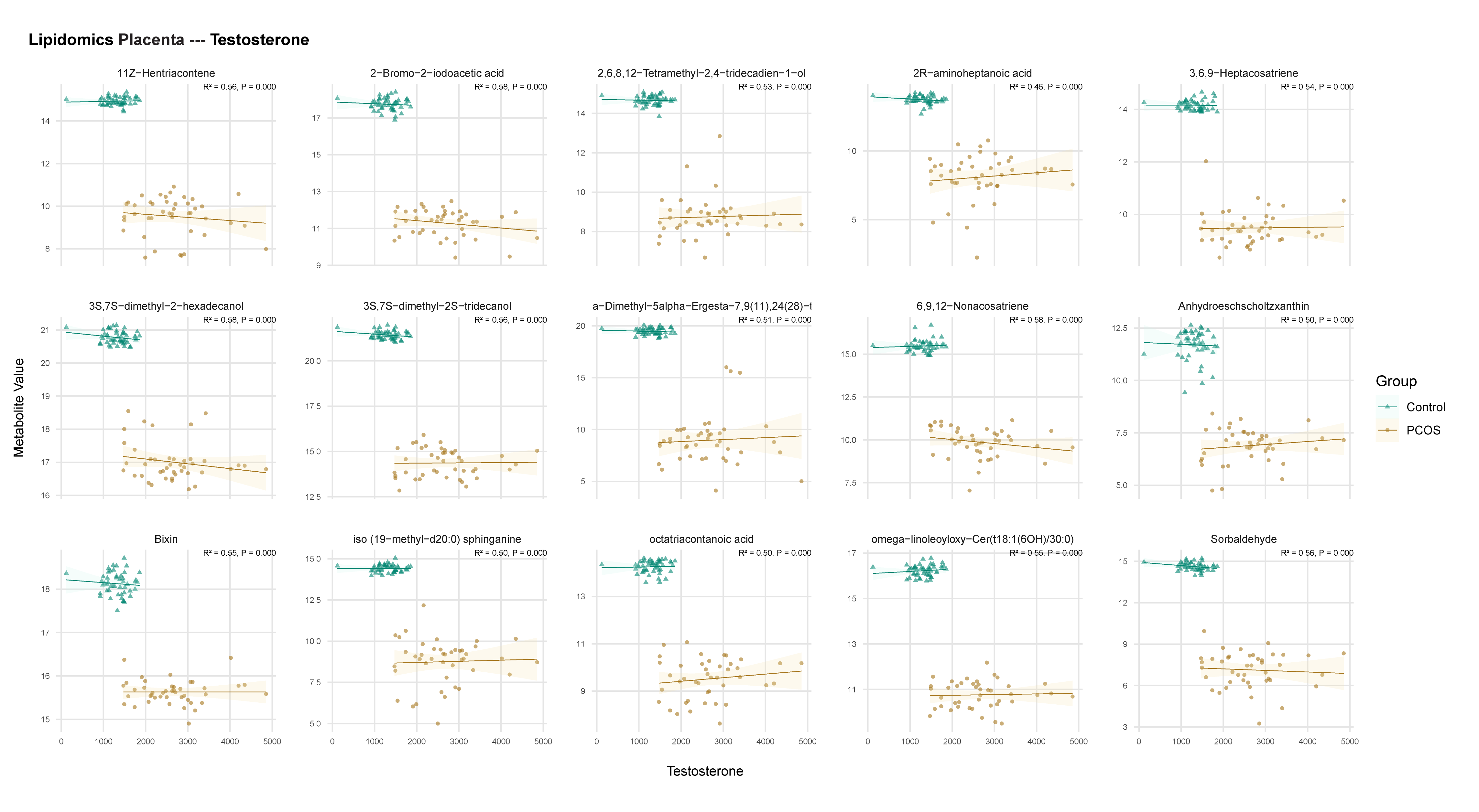


**Supplementary Figure S10.** Correlation analysis of key differential lipids in lipidomics from placental tissue with maternal testosterone levels in PCOS and control groups.


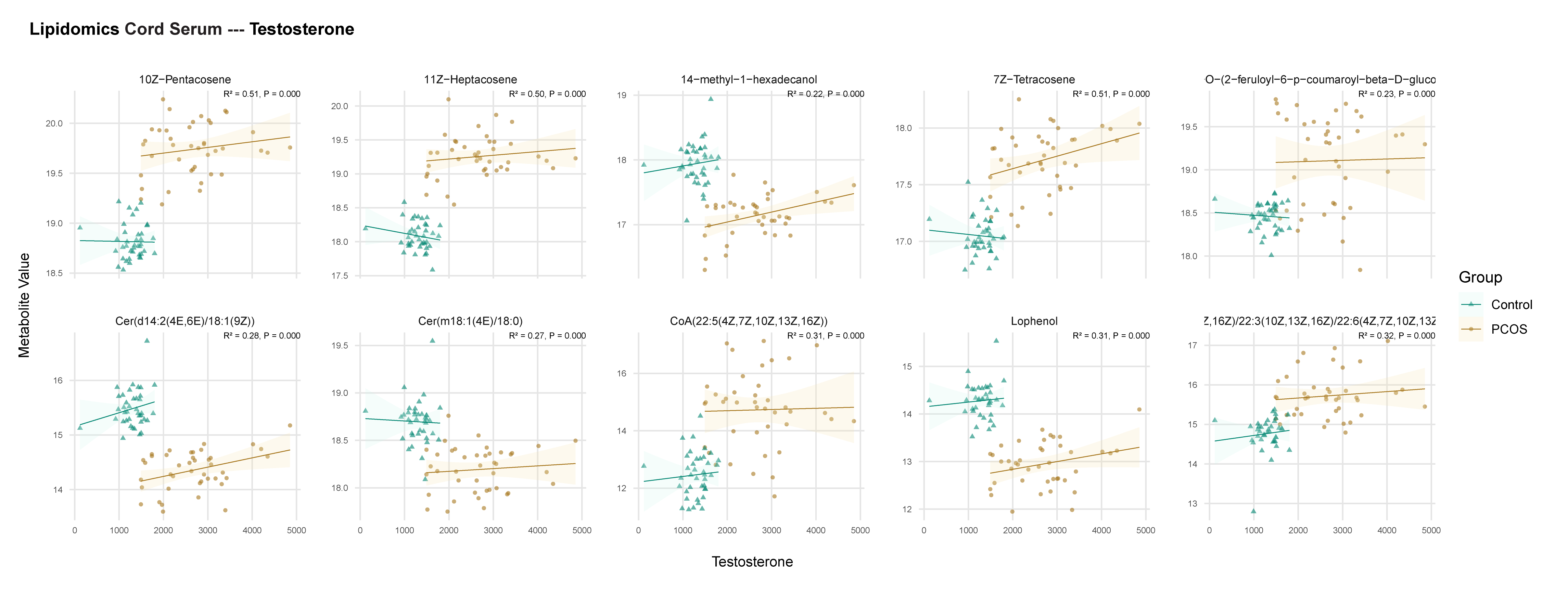


**Supplementary Figure S11.** Correlation analysis of key differential lipids in lipidomics from umbilical cord serum with maternal testosterone levels in PCOS and control groups.


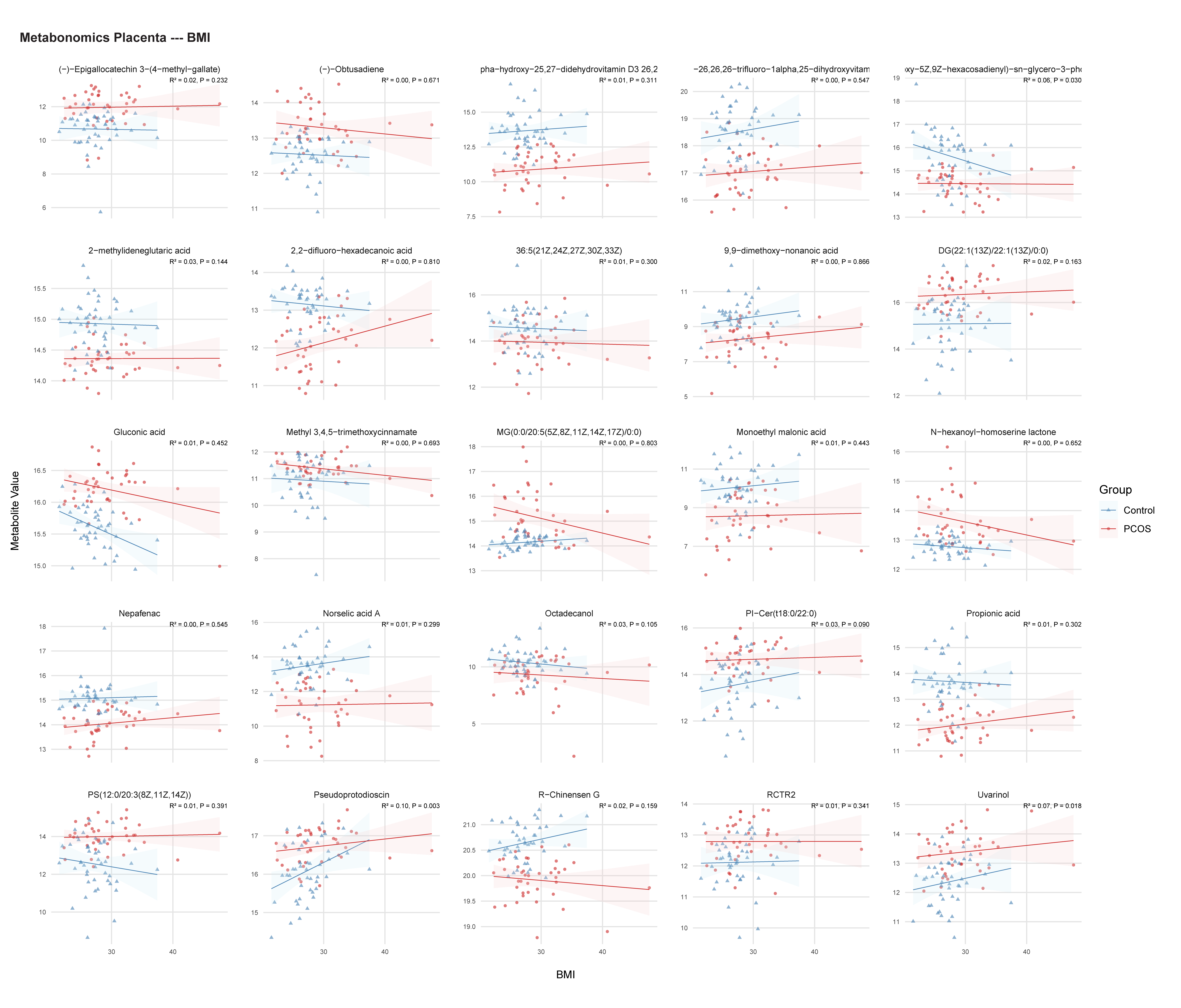


**Supplementary Figure S12.** Correlation analysis of key differential metabolites in metabolomics from placental tissue with maternal BMI in PCOS and control groups.


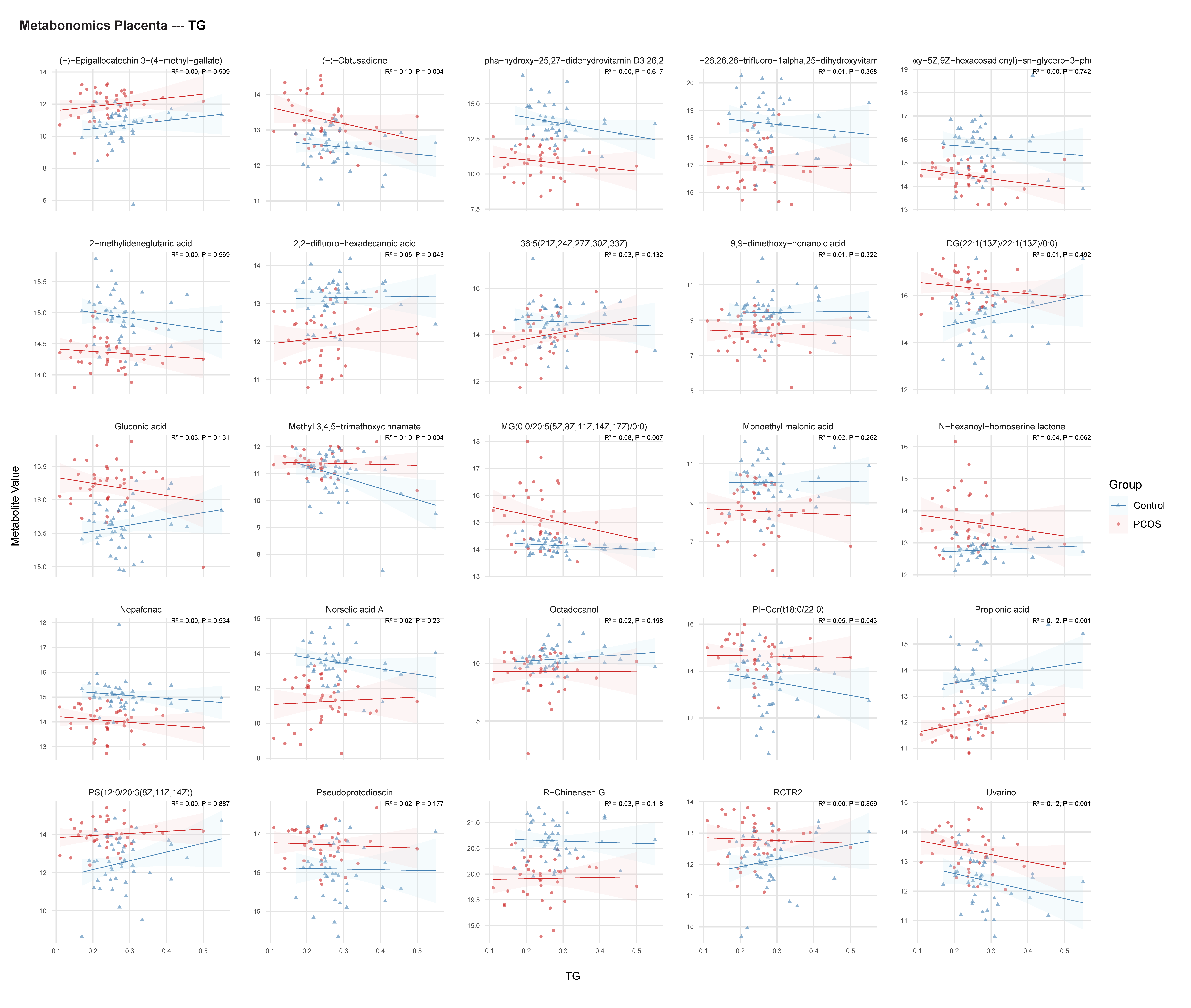


**Supplementary Figure S13.** Correlation analysis of key differential metabolites in metabolomics from placental tissue with umbilical cord serum triglycerides (TG) levels in PCOS and control groups.


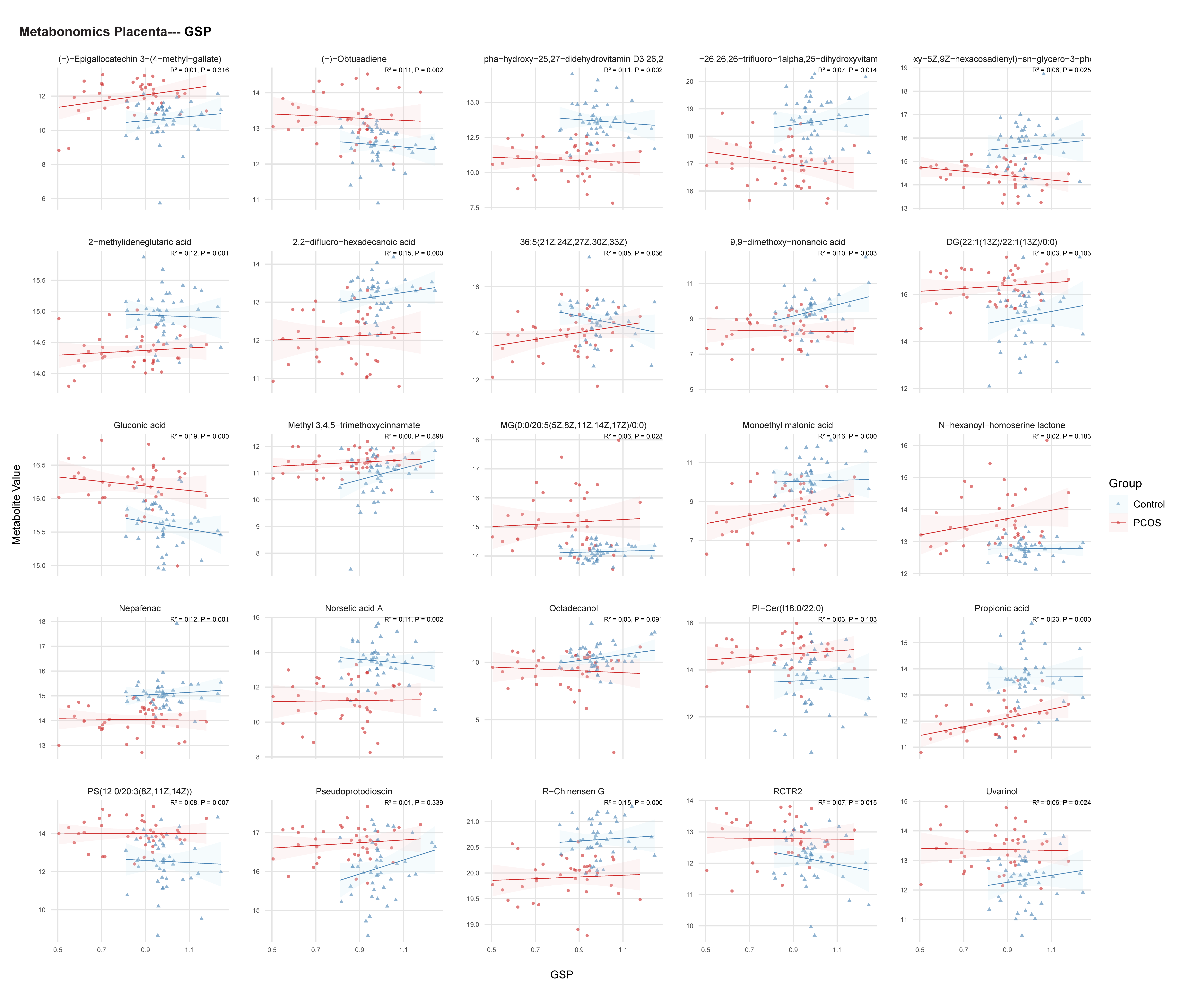


**Supplementary Figure S14.** Correlation analysis of key differential metabolites in metabolomics from placental tissue with umbilical cord serum glycated serum protein (GSP) levels in PCOS and control groups.


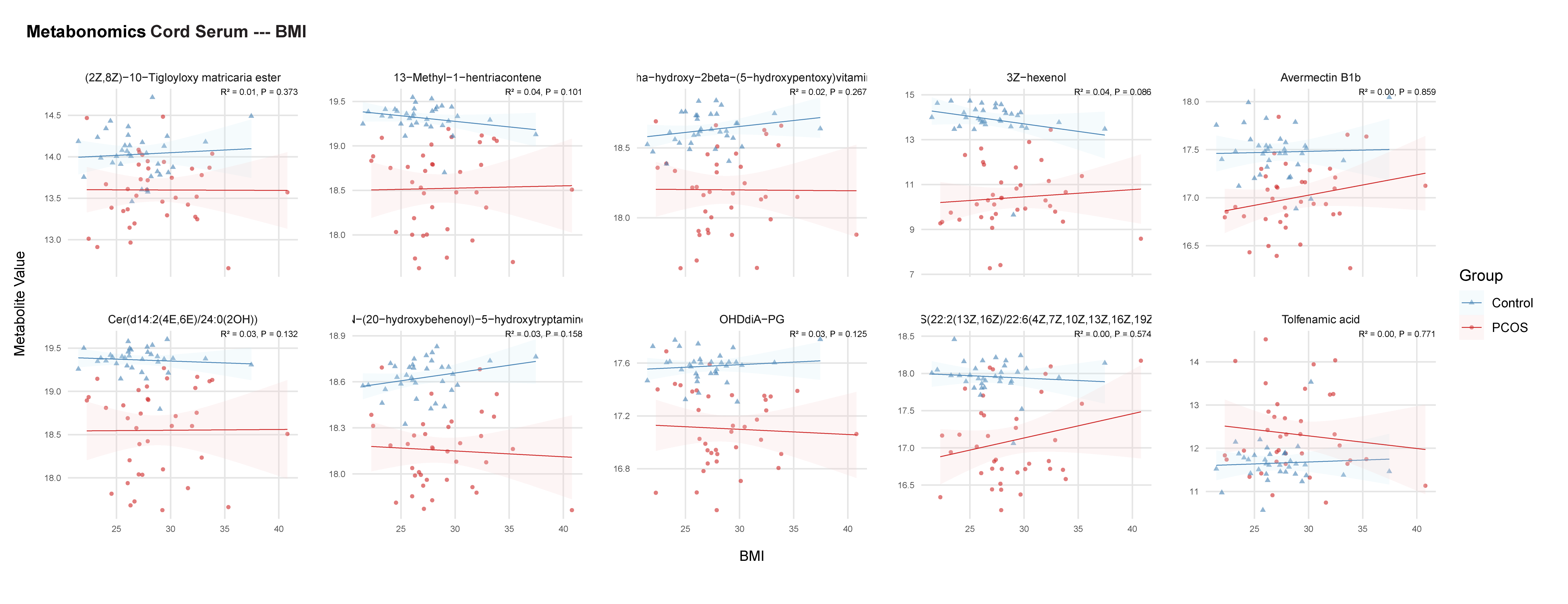


**Supplementary Figure S15.** Correlation analysis of key differential metabolites in metabolomics from umbilical cord serum with maternal BMI in PCOS and control groups.


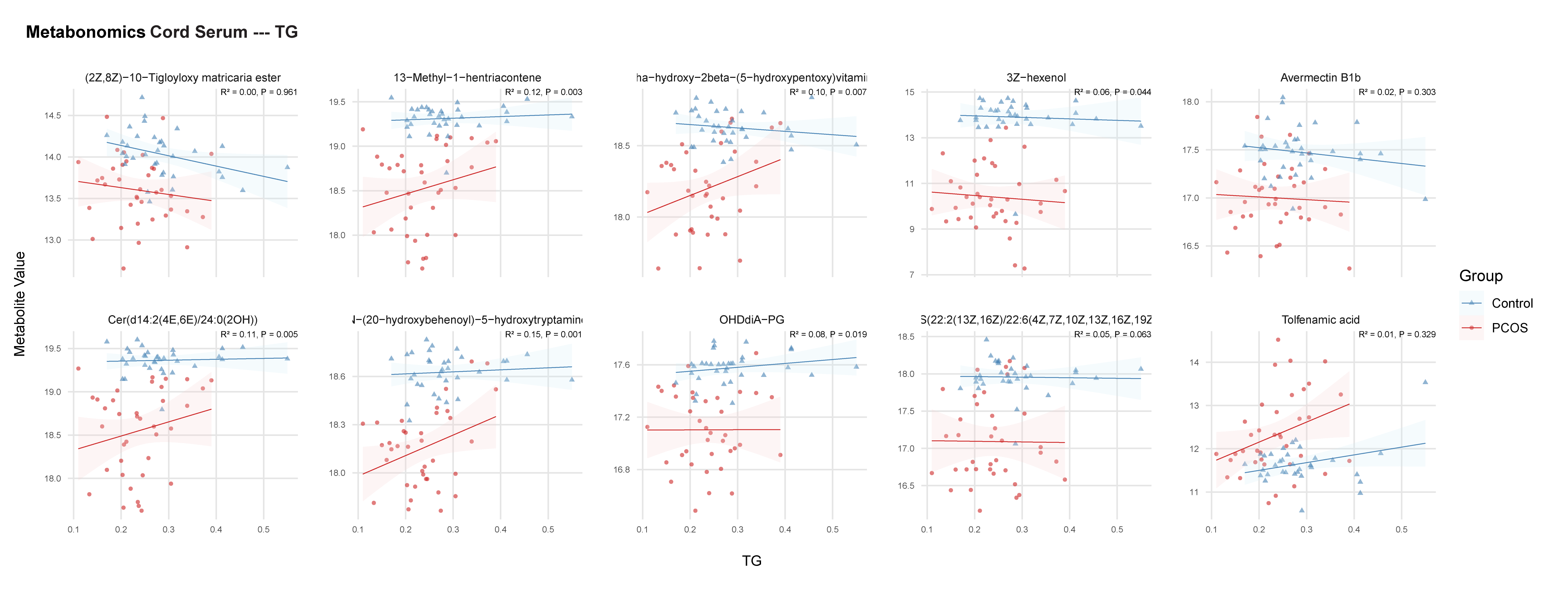


**Supplementary Figure S16.** Correlation analysis of key differential metabolites in metabolomics from umbilical cord serum with umbilical cord serum triglycerides (TG) levels in PCOS and control groups.


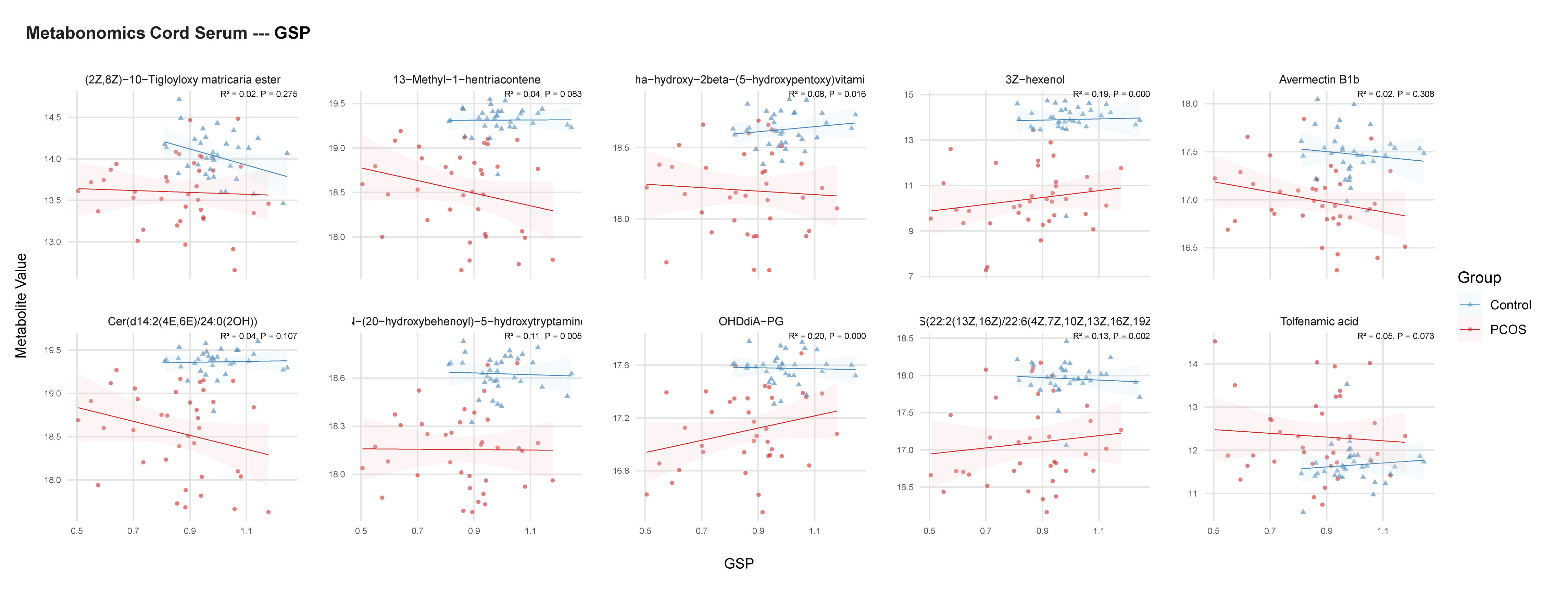


**Supplementary Figure S17.** Correlation analysis of key differential metabolites in metabolomics from umbilical cord serum with umbilical cord serum glycated serum protein (GSP) levels in PCOS and control groups.


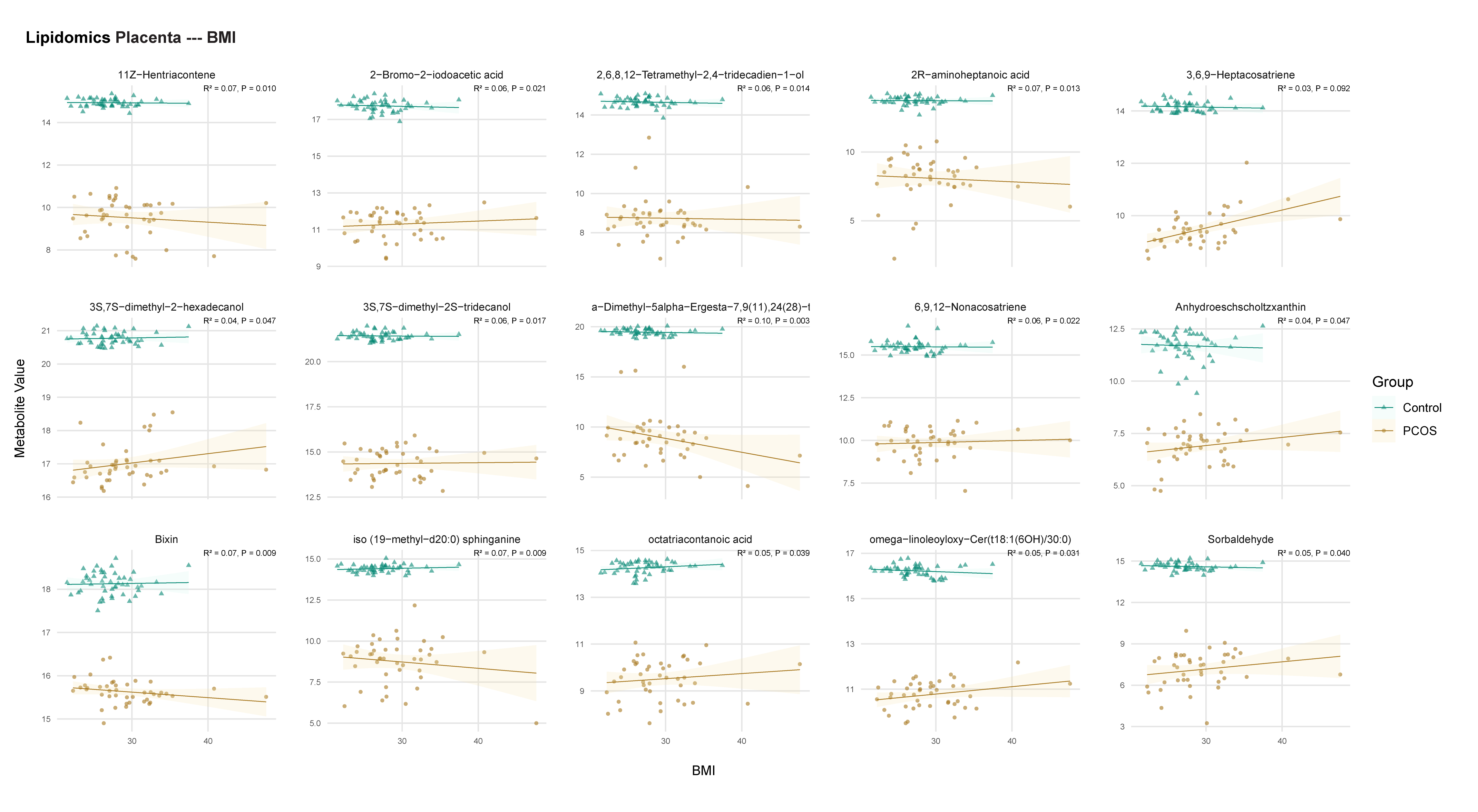


**Supplementary Figure S18.** Correlation analysis of key differential lipids in lipidomics from placental tissue with maternal BMI in PCOS and control groups.


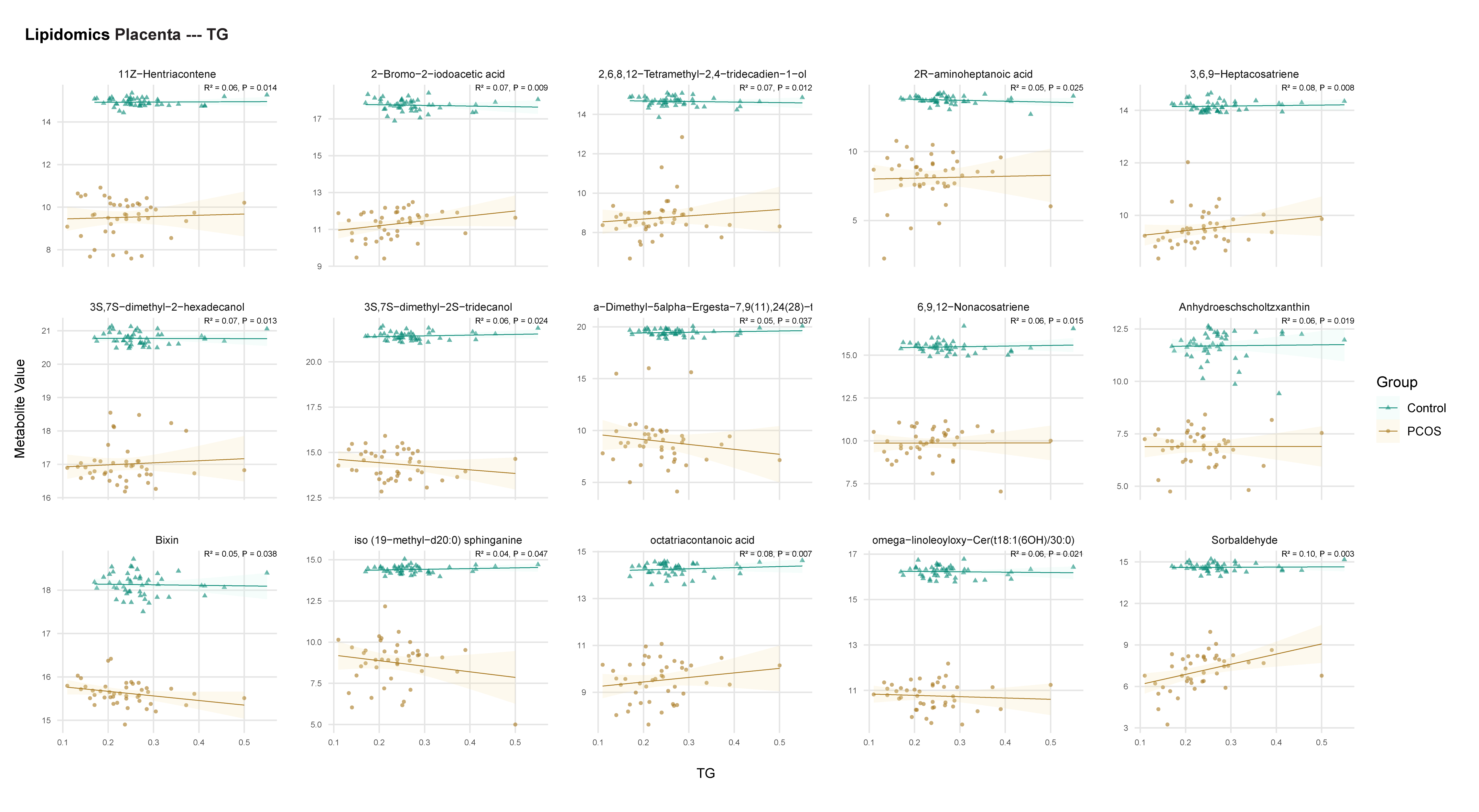


**Supplementary Figure S19.** Correlation analysis of key differential lipids in lipidomics from placental tissue with umbilical cord serum triglycerides (TG) levels in PCOS and control groups.


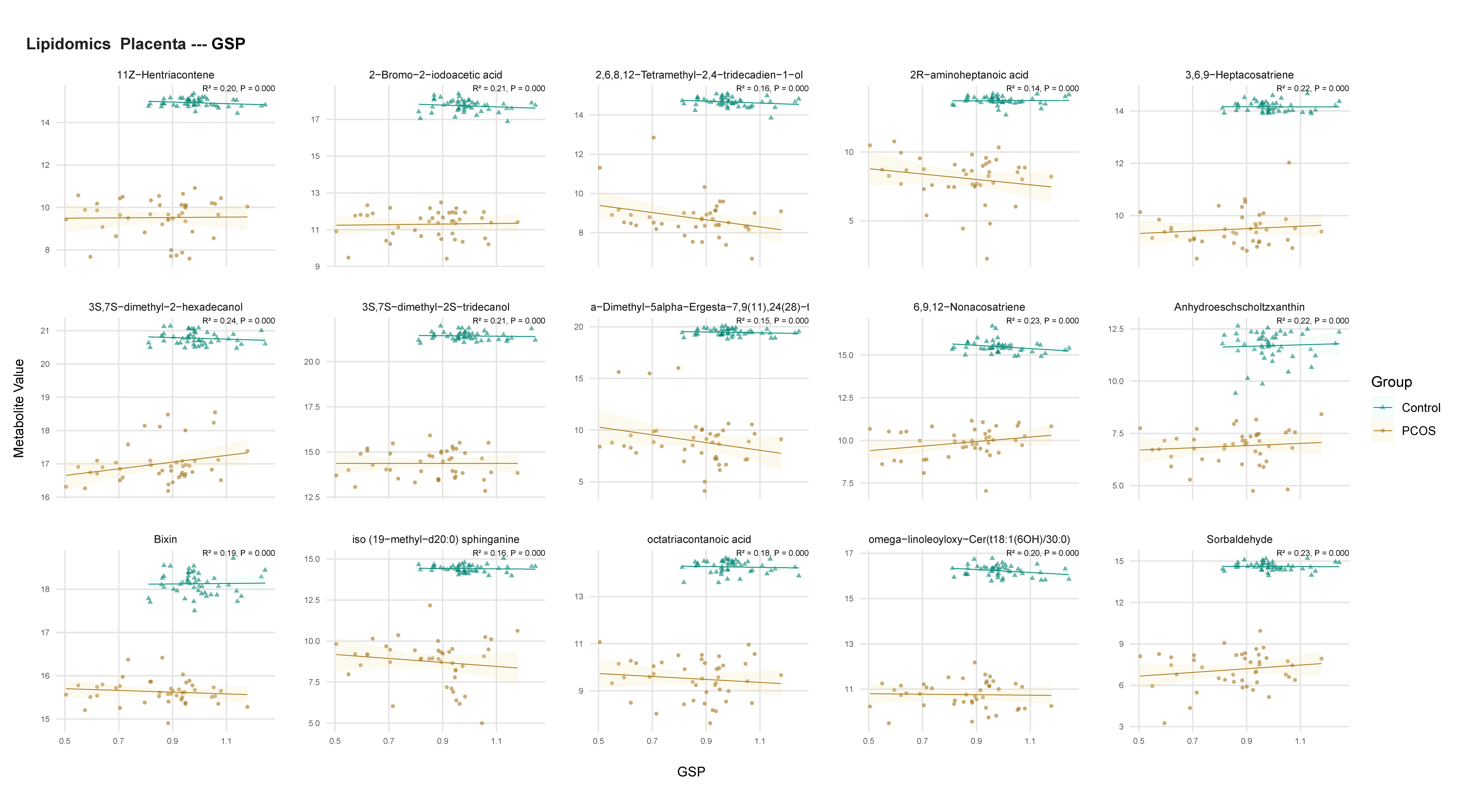


**Supplementary Figure S20.** Correlation analysis of key differential lipids in lipidomics from placental tissue with umbilical cord serum glycated serum protein (GSP) levels in PCOS and control groups.


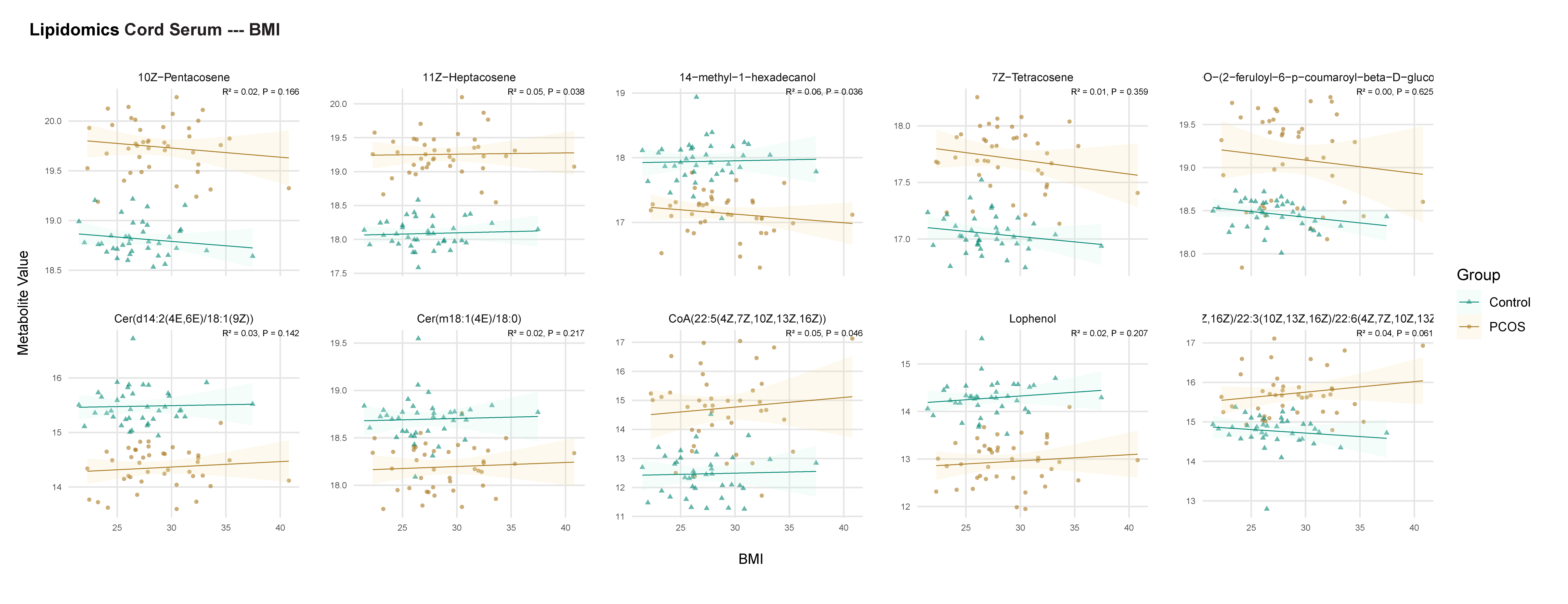


**Supplementary Figure S21.** Correlation analysis of key differential lipids in lipidomics from umbilical cord serum with maternal BMI in PCOS and control groups.


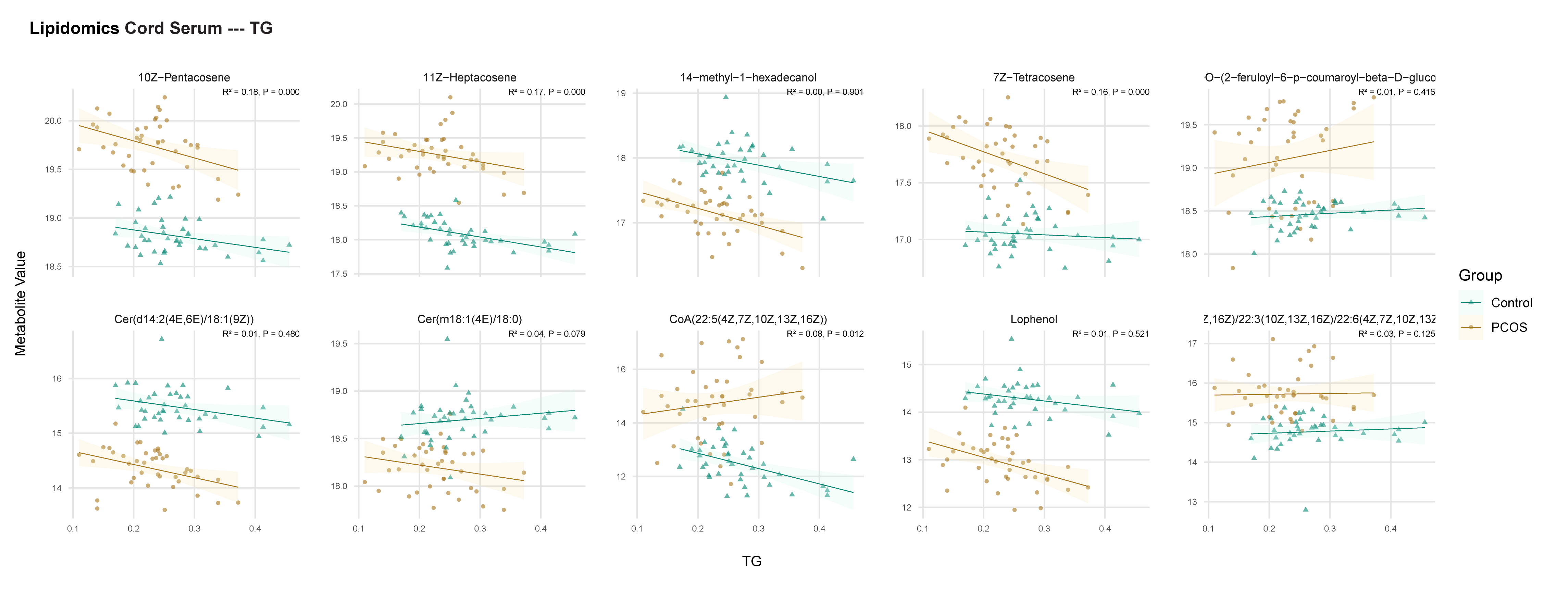


**Supplementary Figure S22.** Correlation analysis of key differential lipids in lipidomics from umbilical cord serum with umbilical cord serum triglycerides (TG) levels in PCOS and control groups.


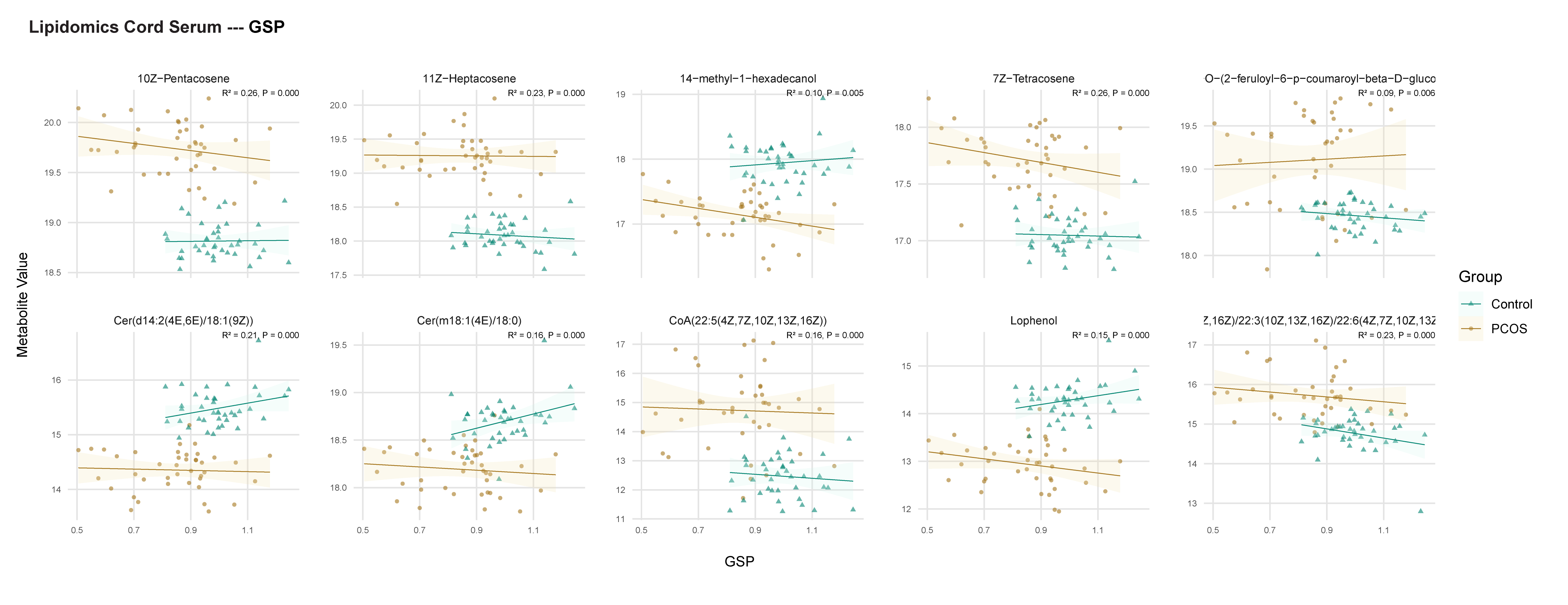


**Supplementary Figure S23.** Correlation analysis of key differential lipids in lipidomics from umbilical cord serum with umbilical cord serum glycated serum protein (GSP) levels in PCOS and control groups.


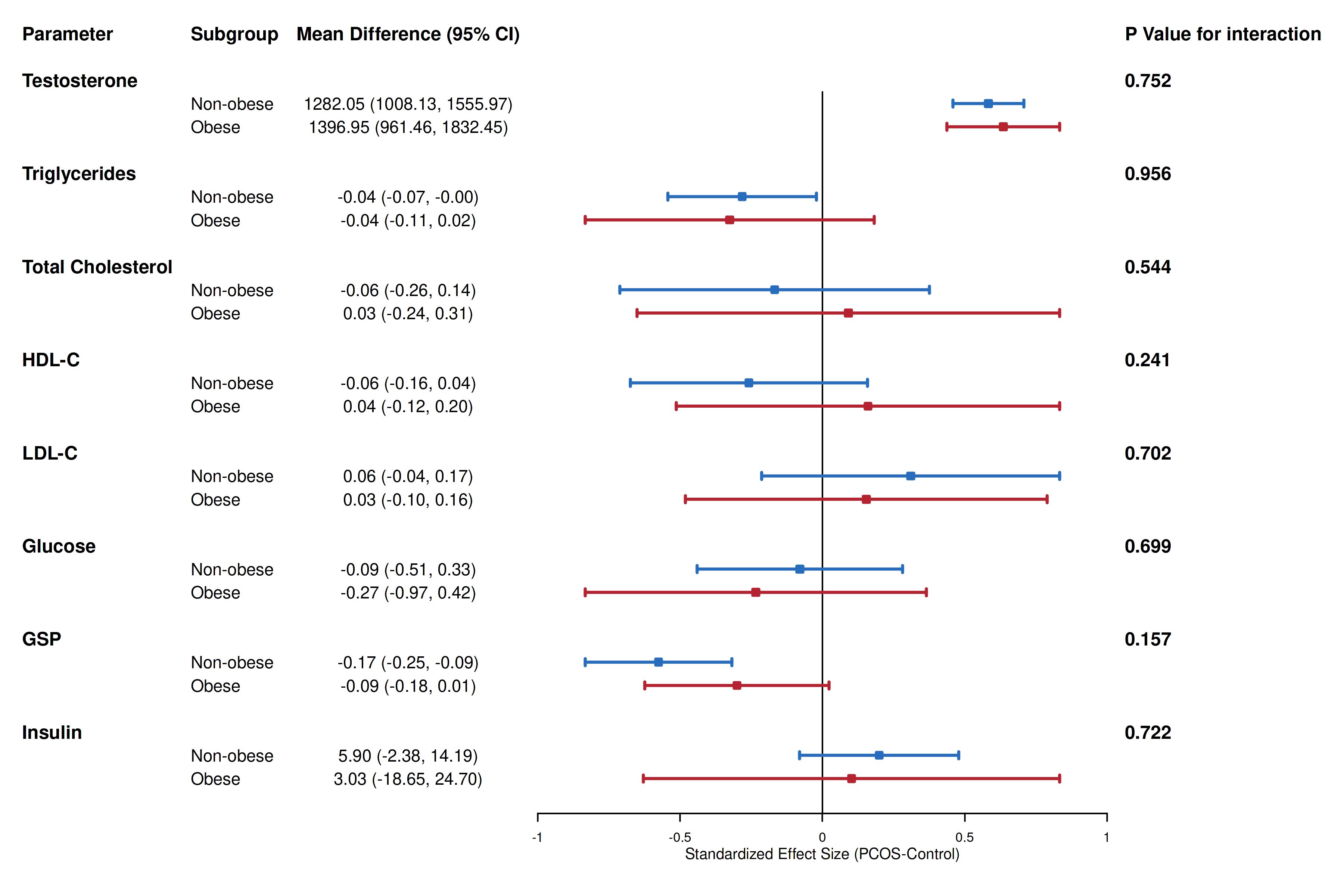


**Supplementary Figure S24. Interaction effects of PCOS and maternal obesity on clinical phenotypes.** Forest plots illustrating the effect sizes (standardized coefficients) of PCOS (vs. Control) on maternal clinical parameters (testosterone) and umbilical cord serum biochemical parameters (triglycerides, total cholesterol, HDL-C, LDL-C, glucose, GSP, insulin), stratified by maternal BMI status (Non-obese vs. Obese). HDL-C, high-density lipoprotein cholesterol; LDL-C, low-density lipoprotein cholesterol; GSP, glycated serum protein.


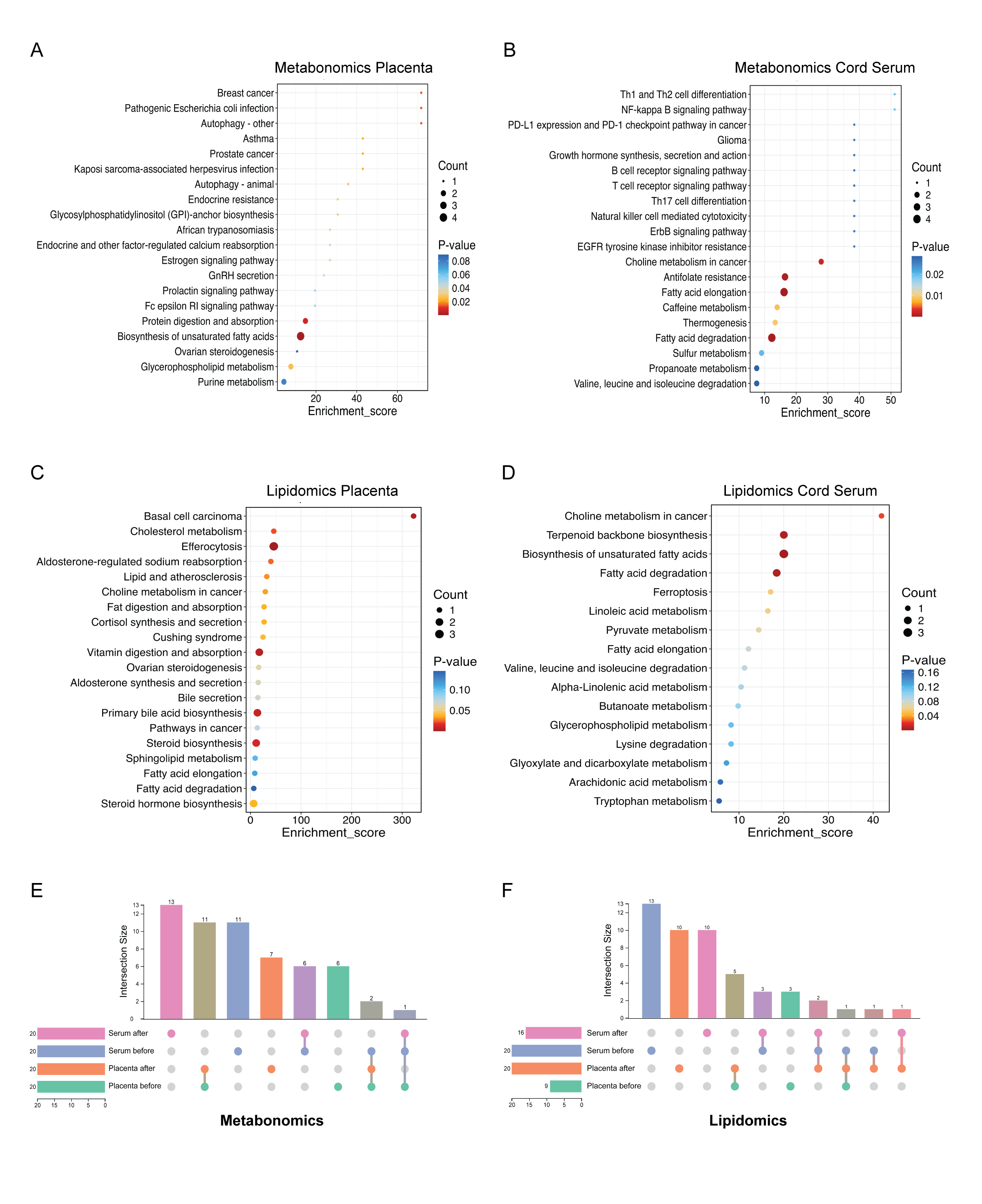


**Supplementary Figure S25.** **Enrichment analysis of differential features between non-obese PCOS and non-obese control subgroups.** (A-B) KEGG enrichment bubble plots of differential metabolites from (A) placental tissue and (B) umbilical cord serum; (C-D) KEGG enrichment bubble plots of differential lipids from (C) placental tissue and (D) umbilical cord serum; (E-F) Upset plots comparing the intersection of enriched KEGG terms from (E) metabolomics and (F) lipidomics datasets before and after BMI stratification.
